# *In Situ* Proinflammatory Effects of Dazostinag Alone or with Chemotherapy on the Tumor Microenvironment of Patients with Head and Neck Squamous Cell Carcinoma

**DOI:** 10.1158/2767-9764.CRC-25-0314

**Published:** 2025-07-30

**Authors:** Richard C. Gregory, Neil Lineberry, Alex Parent, Karthik Rajasekaran, Thomas J. Ow, Cherie-Ann Nathan, Beryl A. Hatton, Wendy Jenkins, Marc Grenley, Connor Burns, Angela Merrell, Jason P. Frazier, Jonathan M.J. Derry, Emily Beirne, Richard A. Klinghoffer

**Affiliations:** 1 Takeda Development Center Americas, Inc. (TDCA), Cambridge, Massachusetts.; 2 University of Pennsylvania, Philadelphia, Pennsylvania.; 3 Albert Einstein College of Medicine and Montefiore Medical Center, Bronx, New York.; 4 Louisiana State University Health, Shreveport, Louisiana.; 5 Presage Biosciences, Inc., Seattle, Washington.

## Abstract

**Purpose:**

The tumor microenvironment (TME) is difficult to model in an *in vivo* cancer research setting. This study leveraged intratumor microdosing using comparative *in vivo* oncology (CIVO) with spatial profiling to evaluate the effects of the stimulator of interferon genes agonist dazostinag, alone or with chemotherapy, on cellular responses within the native TME of intact human tumors.

**Patients and Methods:**

This phase 0 study enrolled adult patients with head and neck squamous cell carcinoma (HNSCC) planned for surgical intervention. Intratumoral microdose injections of dazostinag (maximum dose: 1.68 μg in a 0.05 mg/mL solution), alone or combined with various chemotherapies, were delivered via CIVO to tumors 24, 48, 72, or 96 hours prior to resection. Each tumor sample was prepared for analysis using IHC and ISH. Analysis of the microdosed tumors using the GeoMx Digital Spatial Profiler and CosMx Spatial Molecular Imager was performed in one patient.

**Results:**

Type 1 IFN signaling was induced with dazostinag alone and in combination with chemotherapy from multiple cell types within the TME, including immune cells. Dazostinag also shifted the polarization of macrophages from an immune-suppressive phenotype to a proinflammatory phenotype at 24 hours after injection. Enrichment of cytotoxic T cells was observed in regions of localized dazostinag exposure, coinciding with increased chemokine (CXCL9) expression. Based on cleaved caspase-3, an apoptosis marker, dazostinag plus chemotherapy increased cellular apoptosis relative to either drug alone.

**Conclusions:**

Utilizing CIVO and spatial profiling technology, dazostinag alone and combined with chemotherapy promoted an early proinflammatory response and enhanced chemotherapy-mediated cell death in the native TME of intact human HNSCC tumors.

**Significance:**

The CIVO approach demonstrates that dazostinag alone and combined with chemotherapy promotes both proapoptotic and early proinflammatory responses in the native TME of intact human HNSCC tumors, providing clinical evidence for an on-target mechanism of action and rationale for further clinical investigation.

## Introduction

Dazostinag (TAK-676) is a novel, synthetic stimulator of interferon genes (STING) agonist optimized for systemic delivery that ignites the innate immune system and mobilizes adaptive immunity (1). In syngeneic mouse models, treatment of tumor-bearing mice with dazostinag promoted innate and adaptive immune responses via activation of dendritic cells, NK cells, and CD8^+^ T cells within the tumor microenvironment (TME) and associated lymphoid tissue, resulting in antitumor responses (1, 2). Dazostinag is under investigation as monotherapy, in combination with immune checkpoint inhibitors (ICI) and chemotherapy, and following radiotherapy for patients with locally advanced or metastatic solid tumors (3–7).

The treatment of head and neck tumors, a collection of cancers that originate in the mouth, throat, sinuses, and salivary glands, represents an area of unmet medical need. Dependent on the anatomic site of the tumor, treatment includes combinations of surgery, radiation, chemotherapy, EGFR-targeted therapies, and immunotherapies (8). Although ICI treatment with pembrolizumab was shown to positively affect the survival of patients with head and neck squamous cell carcinoma (HNSCC) in KEYNOTE-048 (9), additional treatments and combinations of therapies are warranted. STING agonism is emerging as a potential mechanism to enhance the response of HNSCC tumors to immune-oncology therapies and chemotherapies. For example, STING activation potentiates an adaptive CD8^+^ T-cell response to treatment with a PD-L1 monoclonal antibody in inflamed HNSCC tumor models (10). Furthermore, treatment of tumors with DNA cross-linking agents (e.g., cisplatin) that result in the production of reactive oxygen species is reported to be dependent on STING expression for maximal activity (11). STING expression has been reported in human papillomavirus–positive (HPV^+^) HNSCC, with host STING activation via cyclic dinucleotides linked to tumor T-cell infiltration and induction of a durable response in murine models (12). Additionally, STING degradation has been identified as a mechanism of immune escape following HPV16 transduction, whereas higher levels of STING expression in patients with HNSCC are linked to a favorable clinical response (13). Data also show that the expression of STING and IFN-β in HPV^+^ tumors is correlated with survival in patient samples (14).

The TME is complex, constantly evolving, and unique to each individual, making it a critical area of research for cancer therapies (15, 16). The TME comprises diverse stromal, immune, and cancer cellular components, which together play essential roles in cancer pathogenesis (15, 16). Tumor cells modify the TME via direct and indirect interactions with immune cells, leading to impaired immune surveillance and tumor immune escape (17). Macrophages are key innate regulators of a productive immune response; however, macrophages within the TME are alternatively activated and support a protumorigenic, immune-suppressive microenvironment via the expression of PD-L1 and the production of immune-suppressive cytokines and chemokines (18, 19). Notably, PD-L1 upregulation has been linked to IFN-γ signaling within the TME as a response to immune activation (20). In this regard, examination of the effects of STING agonism on localized immune responses within the TME of human tumors would provide valuable mechanistic information on dazostinag although this is challenging in a research setting (21). Indeed, in most cases, promising preclinical oncology data with novel therapeutics fail to translate into efficacy in later clinical trials (22, 23).

The comparative *in vivo* oncology (CIVO) platform was developed to address challenges surrounding discordance in data gathered from laboratory models of cancer versus the clinical setting (24, 25). CIVO is a handheld injection device that is used to introduce microdose quantities (i.e., subtherapeutic doses that would not induce systemic toxicity) of up to eight drugs or drug combinations in distinct, trackable locations within the tumor (i.e., in the native and structurally intact TME of human patients with cancer; Supplementary Fig. S1). The injected tissue is then excised to assess drug-induced events, capturing tumor cells and TME responses (24, 25). In a first-of-its-kind phase 0 study, CIVO was combined with two spatial profiling platforms [digital spatial profiling (DSP), GeoMx; spatial molecular imaging (SMI), CosMx] to assess the mechanistic effects of an investigational SUMOylation inhibitor (subasumstat, TAK-981) on the intact, native TME of patients with HNSCC (26); this study revealed the feasibility of using this technology to demonstrate the molecular and cellular effects of subasumstat *in situ*.

Leveraging the CIVO platform and spatial profiling technology, which allows for drug response assessment at the single-cell level in the preserved TME, the present phase 0 study aimed to evaluate the changes induced by the STING agonist dazostinag alone and in combination with standard-of-care chemotherapy, including carboplatin, paclitaxel, and 5-fluorouracil (5-FU) within the TME of patients with resectable HNSCC.

## Patients and Methods

### Study design

A phase 0, open-label, multicenter, unblinded, multiagent, localized, pharmacodynamic biomarker study (NCT04541108) recruited patients with HNSCC from four clinical sites in the United States (University of Pennsylvania, Philadelphia, PA; Albert Einstein College of Medicine and Montefiore Medical Center, Bronx, NY; Louisiana State University Health, Shreveport, LA; and University of Cincinnati Health, Cincinnati, OH) to evaluate the mechanistic effects of microdose injections of dazostinag.

This study was conducted in accordance with the ethical guidelines outlined in the Declaration of Helsinki and the International Council on Harmonisation guidelines on Good Clinical Practice. All clinical sites underwent qualification review and received approval from an Institutional Review Board, and all patients provided informed consent.

### Patients

Eligible patients were aged ≥18 years at the screening visit with a pathologic diagnosis of HNSCC and an Eastern Cooperative Oncology Group performance status of 0 to 2. Patients had to have ≥1 lesion (primary or recurrent tumor, or metastatic lymph node) that measured ≥2 cm in the shortest diameter, for which surgical intervention was scheduled as part of standard care and that was surface-accessible for CIVO injection. The treatment plan could also include adjuvant radiation or chemotherapy. Patients with tumors lacking a sufficient volume of viable tumor tissue for CIVO microdose injections were not eligible for inclusion. The full eligibility criteria are shown in Supplementary Information S1.

### Study objective

The primary objective of this study was to evaluate the effect of dazostinag alone and in combination with chemotherapy, when administered intratumorally using CIVO, on localized immune responses within the TME of patients with HNSCC planned for tumor/regional node dissection as part of the standard of care.

### Treatments and microdosing

To select an appropriate dose of dazostinag, a dose-finding study in syngeneic mice was performed (Supplementary Information S1; Supplementary Figs. S2–S4). This preclinical study found that the intermediate dose of 0.05 mg/mL (0.5 μg) of dazostinag demonstrated the optimum balance between on-target immune cell activation and recruitment while avoiding deleterious off-target effects; it was, therefore, chosen as the optimal dose for future phase 0 studies.

The selected 0.5 μg dose per needle enabled the administration of dazostinag in up to four needles in a single study while meeting the FDA guidance of a maximum microdose (defined as ≤1/100th of the intended clinical dose) of 2 μg per patient (27). Therefore, the maximum amounts of dazostinag delivered per microdose needle and per patient were 0.42 and 1.68 μg, respectively, in a 0.05 mg/mL solution. The chemotherapy agents, that is, carboplatin, paclitaxel, and 5-FU, were appropriately diluted so that the maximum amounts per needle maintained the total amount microdosed per patient below the 100-μg limit. This enabled the evaluation of dazostinag alone and combined with standard-of-care chemotherapy: carboplatin (2 mg/mL), carboplatin (2 mg/mL) + paclitaxel (2.4 mg/mL), and carboplatin (2 mg/mL) + 5-FU (4 mg/mL).

### Assessments

Each eligible patient participated in the study for up to 8 weeks, which consisted of a screening period (days −28 to 0); microdose injection with the CIVO device, configured with three, five, or eight needles depending on lesion dimensions; followed by surgical intervention (days 1–5); and follow-up (one visit between days 6 and 14 and the final visit on day 28). Adverse events were monitored from initial enrollment until study completion on day 28.

HPV status was determined via routine clinical testing and reported by the respective participating centers. Reported results were validated via p16 IHC staining, which was in agreement with clinical testing in all available cases (*n* = 10); some cases were not tested/reported by the clinical centers (*n* = 5). For these, only laboratory-based p16 staining information was available. We report HPV status based on laboratory testing.

CIVO microdose injections of dazostinag alone or combined with chemotherapy were delivered at 24, 48, 72, or 96 hours prior to tumor resection to assess early and late immune activation responses. Injection sites were visible due to the presence of coinjected fluorescent tracking microspheres (CIVO GLO; refs. 25, 26) in each injection column. For procedures performed with the 3- and 5-needle devices, localized drug-exposed regions were compared with noninjected regions of the tumor, representing no-drug controls. For procedures performed with eight-needle devices, a vehicle (control) injection site was also included, containing only CIVO GLO and 0.9% saline. After resection, each tumor sample was immediately cross-sectioned perpendicular to the site of the microdose injections, formalin-fixed, and shipped to Presage Biosciences where they were paraffin-embedded, cut onto slides with a thickness of 4 μm, stained, and analyzed. The CIVO microdosing procedure has been detailed previously, as have details of biomarker staining using IHC and ISH (24–26). Primary and secondary antibodies used in the IHC assays are listed in Supplementary Table S1; whole slide images were acquired at 20× magnification using a 3DHISTECH Pannoramic 250 Flash digital slide scanner (3DHISTECH) or ZEISS Axioscan 7 (ZEISS; RRID: SCR_020927). Probes used for ISH are detailed in Supplementary Table S2. Analysis of the microdosed tumors with spatial profiling, using both DSP (GeoMx; RRID: SCR_021660) and SMI (CosMx; SCR_023909), was also performed in one patient. The methodology for spatial profiling and imaging has been described in detail previously (26) and can be found in Supplementary Information S1.

### Statistical analysis

Clinical data metrics were collected on the Medidata Rave Electronic Data Capture platform. Data were summarized descriptively for patient demographics and other nonsafety-related clinical data. NanoString DSP and CosMx SMI analyses were performed in RStudio (version 2021.09.0; RRID: SCR_000432) using R version 4.1.1.

### Data availability

The datasets, including the redacted study protocol, redacted statistical analysis plan, and individual participants’ data supporting the results of the completed study, will be made available after the publication of study results within 3 months of the initial request to researchers who provide a methodologically sound proposal. The data will be provided after deidentification, in compliance with applicable privacy laws, data protection, and requirements for consent and anonymization.

## Results

### Patient characteristics and sample selection for analysis

Of the 15 patients enrolled, 14 completed the study, and one patient died due to disease progression prior to the final study visit. Patient demographics and baseline characteristics for all 15 patients are provided in Table 1; the representativeness of patients in the study is shown in Supplementary Table S3. Tumor type/location and HPV and smoking status of each patient are shown in Table 2; 10 of 15 patients were HPV-positive as determined via p16 staining, and 9 of 15 were current or previous smokers.

**Table 1:** Demographics and characteristics of patients enrolled in the study

Parameter	*N* = 15
Age, years	​
Median (min, max)	64 (48, 72)
Location of injected lesion, *n*	​
Oral cavity^a^	6
Cervical lymph node^b^	8
Lip	1
Disease type	​
Localized	8
Metastatic	7
HPV status, *n*	​
Positive	10
Negative	5
Smoking status, *n*	​
Current smoker	3
Previous smoker	6
Never smoked	6
Sex, *n*	​
Male	14
Ethnicity, *n*	​
Not Hispanic or Latina	7
Hispanic or Latina	5
Not reported	2
Unknown	1
Race, *n*	​
White	11
Other	3
Unknown	1

a Tongue (*n* = 3), mandible (*n* = 2), right buccal mucosa (*n* = 1).

b Right cervical lymph node (*n* = 4), left neck lymph node (*n* = 2), left level IIA lymph node (*n* = 1), cervical lymph node (*n* = 1).

**Table 2:** Tumor details and smoking and HPV status of individual patients

Patient number	Disease type	Anatomic site of injected/resected tumor	Smoking status	HPV status (p16 IHC)
1	Local	Oral cavity/tongue	Current	Negative
2	Metastatic	Left level IIA lymph node	Never	Positive
3	Metastatic	Left neck lymph node	Previous	Positive
4	Local	Cervical lymph node	Never	Positive
5	Local	Right cervical lymph node	Never	Positive
6	Local	Left neck lymph node	Previous	Positive
7	Local	Mandible	Previous	Negative
8	Metastatic	Tongue	Previous	Positive
9	Metastatic	Right cervical lymph node	Never	Positive
10	Metastatic	Right cervical lymph node	Current	Positive
11	Local	Left tongue	Never	Positive
12	Metastatic	Left mandible oral lesion	Current	Negative
13	Local	Left lower lip/chin	Previous	Negative
14	Metastatic	Right cervical lymph node	Previous	Positive
15	Local	Right buccal mucosa	Never	Negative

Following the CIVO microdose injection, tumor resection was performed after 24 hours in seven patients, 48 hours in one patient, 72 hours in five patients, and 96 hours in two patients. Tumor samples from 12 patients exhibited trackable sites of drug microdose injection and were of sufficient tissue quality to be selected for further analysis. Subsequent biomarker analysis was completed in 10 samples from multiple injection sites within the same sample (four samples from the 24-hour cohort and six samples from the 72- to 96-hour cohort). As data from the late (≥72-hour) cohort showed that levels of various biomarkers were transient and had returned to baseline levels upon examination, results will focus on the 24-hour cohort. The CIVO microdosing procedure was well tolerated with no adverse events deemed related to the injection procedure or its contents.

### Dazostinag induced a type 1 IFN response as detected by GeoMx DSP and confirmed by ISH

#### Spatial profiling revealed remodeling of the TME following dazostinag exposure

**Figure 1:**
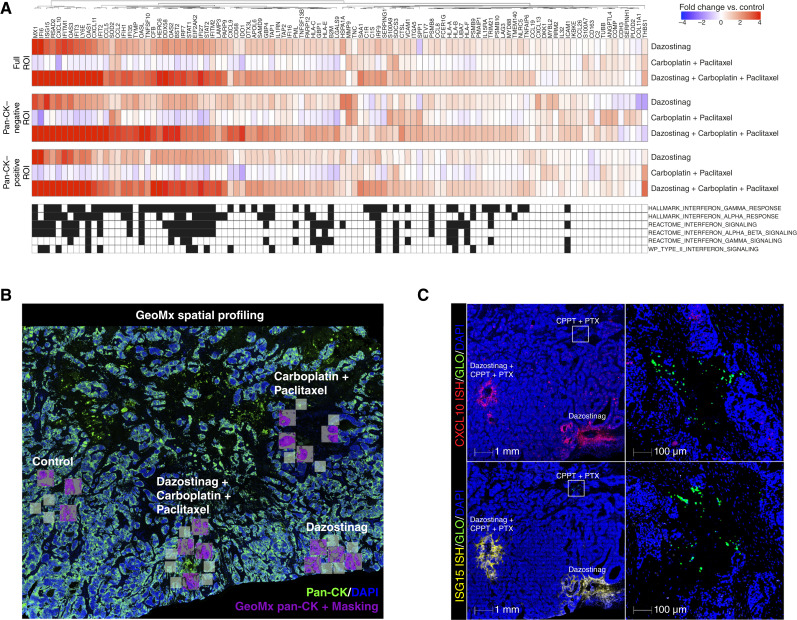
Dazostinag induces a type 1 IFN response detected by (**A** and **B**) GeoMx digital spatial profiling and (**C**) by ISH. The 104 genes in the dazostinag signature (**A**) were analyzed for enrichment in biological pathways using the Molecular Signatures Database (Broad Institute, Cambridge, MA; RRID: SCR_016863). The annotation below the heatmap shows very high enrichment in IFN pathways: HALLMARK_INTERFERON_GAMMA_RESPONSE fourfold enrichment, *P* = 2.61E^−91^; HALLMARK_INTERFERON_ALPHA_RESPONSE threefold enrichment, *P* = 2.66E^−65^; REACTOME_INTERFERON_SIGNALING sevenfold enrichment, *P* = 7.96E^−52^; REACTOME_INTERFERON_ALPHA_BETA_SIGNALING threefold enrichment, *P* = 3.17E^−49^; REACTOME_INTERFERON_GAMMA_SIGNALING fivefold enrichment, *P* = 1.24E^−30^; WP_TYPE_II_INTERFERON_SIGNALING twofold enrichment, *P* = 2.69E^−30^. **A** and **B**, Spatial profiling of >1,800 gene targets was performed at sites of microdose exposure for a 24-hour sample in one patient. Both pan-CK–masked areas (shown as purple and gray areas of larger squares on **B**) and unmasked areas (shown as smaller gray squares on **B**) were collected, and response heatmaps were analyzed by mask. Treatment effect was reported as the fold change over the vehicle control injection site. Genes positively associated with dazostinag exposure are strongly correlated with an induction of IFN signaling and are shown in red; those enriched in the downregulated gene set are shown in blue. **C,** Representative images of drug injection sites showing enrichment of IFN genes, including *Cxcl10* (red) and *Isg15* (yellow), after 24 hours of dazostinag monotherapy, CPPT + PTX, and dazostinag + CPPT + PTX exposure (left) and 20× magnification of the CPPT + PTX injection site (right). Cell nuclei are stained with DAPI (blue), and CIVO GLO is shown in green. CPPT, carboplatin; PTX, paclitaxel; ROI, region of interest.

To assess the ability of dazostinag to remodel the TME via induction of IFN signaling, NanoString GeoMx digital spatial profiling of ∼1,800 target genes from one patient sample (patient 2; lymph node tumor, metastatic disease; HPV status, positive; smoking status, never) at 24 hours after intratumoral injection was applied (Supplementary Information S1). This analysis showed that upregulated genes were highly associated with the IFN response pathway at microdose injection sites exposed to dazostinag alone or in combination with chemotherapy (Fig. 1A). By contrast, sites exposed to chemotherapy alone were not associated with increased IFN signaling. Furthermore, the application of a pan-cytokeratin (CK) mask enabled evaluation of gene signatures produced in tumor epithelium (CK+) versus tumor stroma (CK−). The IFN response upregulated at sites exposed to dazostinag was similar between both regions, suggesting that this drug upregulates IFN signaling across multiple cell types (Fig. 1B).

#### Dazostinag exposure is associated with increased type 1 IFN signaling

This analysis set out to determine if local exposure to dazostinag would trigger activation of the STING pathway and expression of type 1 IFN in tumors by using IHC staining for PD-L1 and phospho-IFN regulatory factor 3 and ISH with probes targeting IFN-β1, the ubiquitin-like protein IFN-stimulated gene 15 (ISG15), and the inflammatory cytokines CXCL9 and CXCL10.

IFN-β1 expression was elevated relative to background at sites that had exposure to dazostinag as a single agent in three of four patients evaluated in the 24-hour cohort (Supplementary Fig. S5). All three patients were HPV-positive: two had metastatic disease (patients 2 and 3) and one had local disease (patient 5), and all samples had been resected from lymph nodes (Table 2). The largest overall increase in IFN-β1 was seen for dazostinag + carboplatin + paclitaxel combination sites. Levels of ISG15 were also elevated in all tumors exposed to dazostinag as monotherapy and when combined with chemotherapy. However, no changes relative to background were observed for the chemotherapy-alone treatment arms. Modest increases in PD-L1 with dazostinag alone were seen, with the largest increases occurring with dazostinag combined with carboplatin and 5-FU; by contrast, no significant increases in PD-L1 occurred with chemotherapy alone. To confirm that dazostinag elevates type 1 IFN signaling and actively remodels the TME, the CXCL9-11/CXCR3 axis was examined. ISH data with a probe for the IFN response gene *Cxcl10* showed an increase in its expression levels 24 hours after exposure to dazostinag (Fig. 1C). By contrast, chemotherapy alone failed to induce this effect.

Together, these results confirmed that dazostinag alone or combined with platinum-based chemotherapy stimulates the STING pathways, inducing IFN signaling in tumors 24 hours after intratumoral injection, promoting a proinflammatory TME. The effects on IFN signaling observed at the 24-hour time point were largely abrogated in the late cohort (≥72 hours), with the notable exception of one patient sample (patient 13; lip/chin tumor, local disease; HPV status, negative; smoking status, previous) that had sustained expression of ISG15 at clearly discernible sites exposed to dazostinag alone and in all triple combination sites (Supplementary Fig. S6).

### Multiple cell types within the immune TME are affected by the IFN response as shown by CosMx SMI analysis, including several distinct macrophage subtypes representing different polarity states

#### Cell type–specific responses in the TME associated with an IFN response identified by CIVO plus CosMx SMI

**Figure 2:**
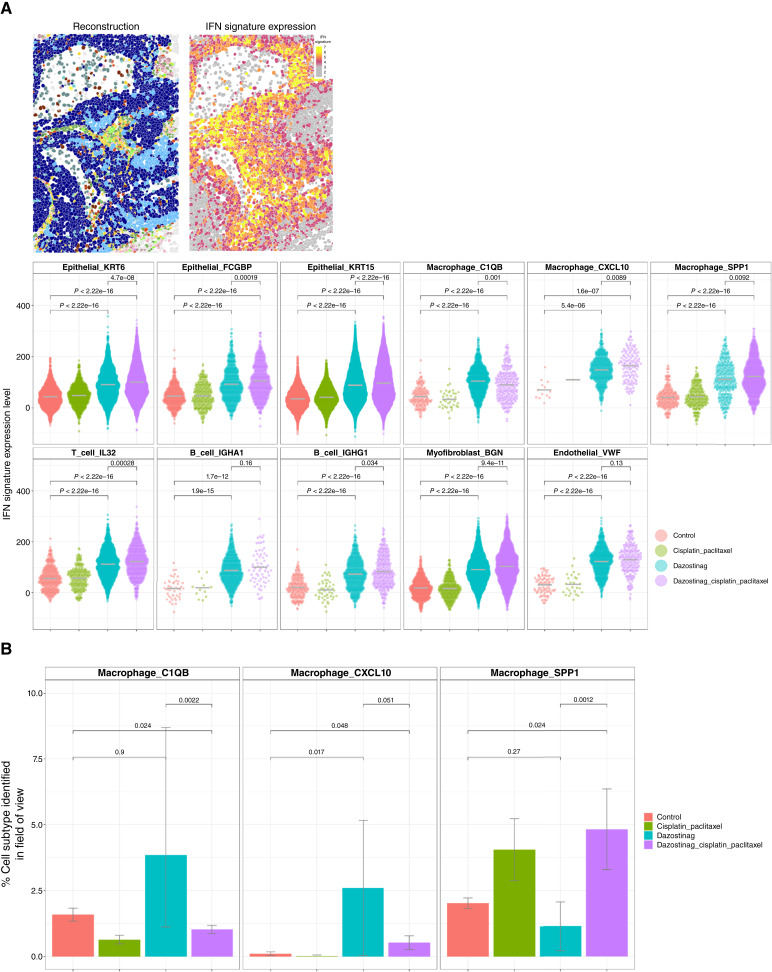
Multiple cell types within the immune TME are affected by the type 1 IFN response as shown by CosMx SMI, including distinct macrophage subtypes of differing polarity. **A,** Spatial reconstruction of the TME. **B,** Macrophage polarization. **A,** The IFN signature associated with a reconstructed region of the TME. **B,** Spatial profiling revealed upregulation of several populations associated with a proinflammatory phenotype, including Macrophage_CXCL10, Macrophage_C1QB, and downregulation of anti-inflammatory Macrophage_SPP1 cells after treatment with dazostinag alone. Combination with chemotherapy maintained a more immune-suppressive TME.

To examine the effects of dazostinag in the TME at single-cell resolution, spatial multiomics with CosMx SMI was performed on a sample from patient 2 at 24 hours after intratumoral injection. We obtained data from multiple drug-exposed and untreated regions throughout the tissue section at 19 independent fields of view (Supplementary Fig. S7). After filtering, 64,525 cells were identified and retained for processing, and categorization was performed using established lineage gene markers with a nonlinear dimensionality reduction technique termed Uniform Manifold Approximation and Projection. Eighteen distinct cell types associated with the TME were identified (including epithelial cells, macrophages, T and B cells, mast cells, and fibroblasts; Supplementary Fig. S7). Spatial reconstruction of the TME showed a strong correlation between dazostinag exposure and induction of IFN expression (Fig. 2A). In agreement with the CK-masked GeoMx DSP results, signaling was increased in dazostinag-exposed regions relative to background and chemotherapy doublets across multiple immune (T-cell, B-cell, macrophage), stromal (myofibroblast, endothelial), and epithelial cell populations. Spatial profiling also demonstrated upregulation of several cellular populations associated with a proinflammatory phenotype (CXCL10) and downregulation of anti-inflammatory macrophage SPP1 with dazostinag alone (Fig. 2B).

#### IHC analysis demonstrated that dazostinag shifts the TME from an immune-suppressive phenotype to a more proinflammatory state 24 hours after intratumoral injection

Ratio shifts in M1- and M2-like macrophages provide insight into the interplay between the host immune system and tumor cells in response to treatment (28). This analysis, therefore, sought to determine the effect of dazostinag by performing IHC analysis with antibodies for CD68, which is ubiquitously expressed by macrophages; CD86 to visualize M1-like proinflammatory macrophages; and CD163 as a biomarker of anti-inflammatory/immune-suppressive M2-like macrophages.

**Figure 3:**
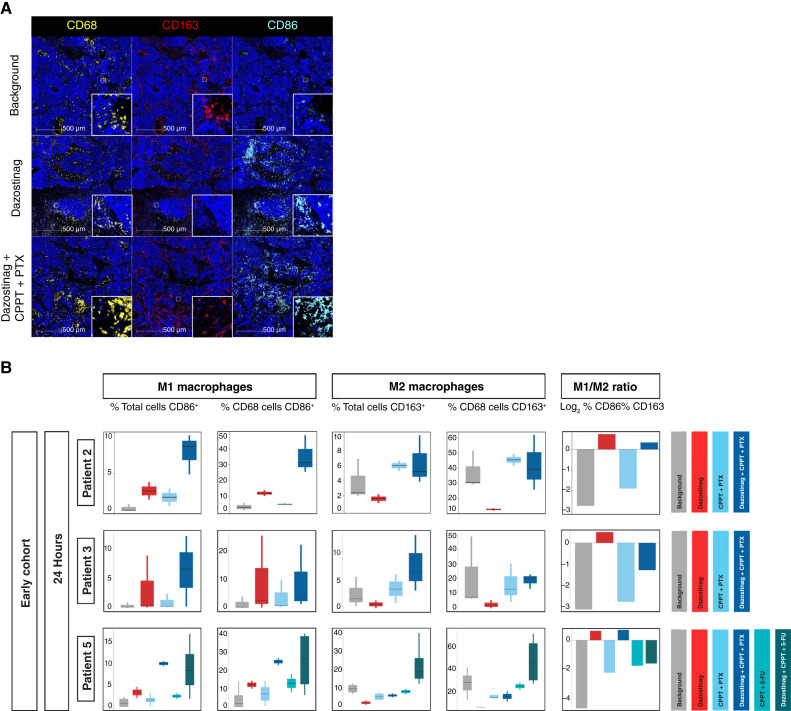
The effect of dazostinag on macrophage polarization was further confirmed by IHC analysis. **A,** Treatment with dazostinag results in a more proinflammatory M1-like phenotype. **B,** Quantification of CD68^+^, CD163^+^, and CD86^+^ macrophages. The number of regions of interest analyzed for each patient and drug condition is detailed in Supplementary Table S4. CPPT, carboplatin; PTX, paclitaxel.

Overall, dazostinag was found to shift the polarization of macrophages from an immune suppressive phenotype to a proinflammatory phenotype at 24 hours after intratumoral injection (Fig. 3A and B). At injection sites that were exposed to dazostinag alone, an increase relative to background was observed in proinflammatory CD86^+^ M1-like macrophages and a decrease in anti-inflammatory CD163^+^ M2-like macrophages across all patient samples at 24 hours. When dazostinag was administered in combination with carboplatin and paclitaxel or carboplatin and 5-FU, the ratio of M1/M2-like macrophages was consistently lower than when it was administered alone, with an increase in CD163 compared with dazostinag monotherapy (Fig. 3B). Moreover, upregulation of CCL2, a chemokine that has been shown to recruit immune-suppressive myeloid cells (29), was observed at injection sites exposed to dazostinag with chemotherapy or chemotherapy alone (Supplementary Fig. S8). Corresponding with GeoMx data, the majority of CCL2 seemed to be expressed by stromal cells rather than tumor epithelium.

### Dazostinag induces CXCL9 signaling and the recruitment of cytotoxic T cells, which ultimately leads to tumor cell death

**Figure 4:**
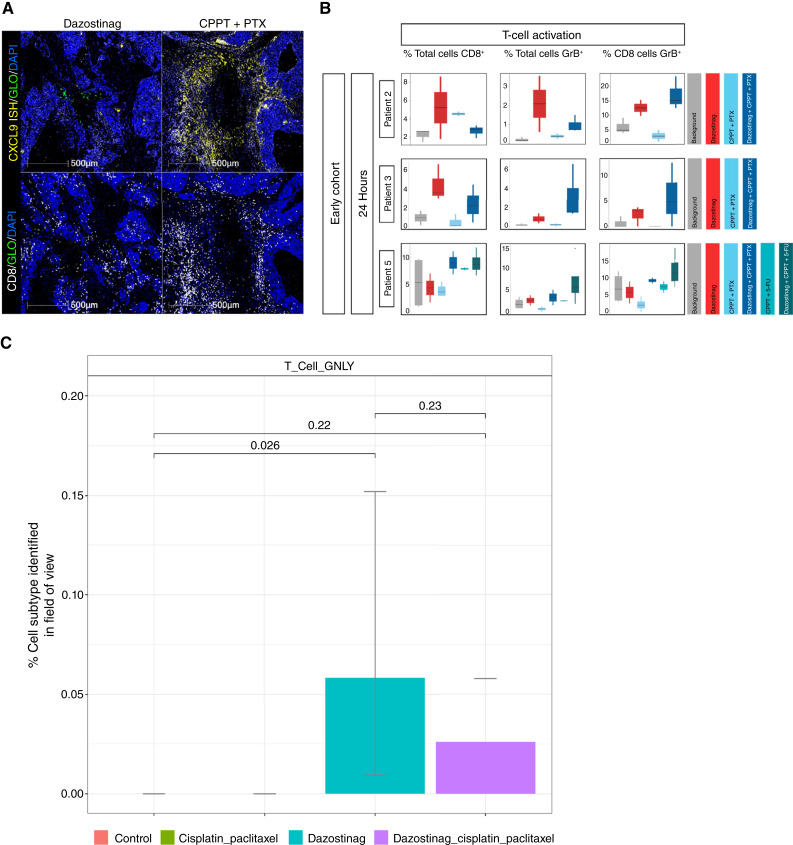
Dazostinag induces CXCL9 signaling and the recruitment of cytotoxic T cells. **A,** Representative images of a chemotherapy doublet site showing no changes in CXCL9 signaling or recruitment of T cells (left) and the dazostinag injection site surrounded by a characteristic ring of drug exposure demonstrating the induction of CXCL9 signaling and recruitment of CD8^+^ T cells (right). **B,** Quantification of T-cell activation. **C,** CosMx data showing increased numbers of T_Cell_GNLY cells that are diminished by combination with chemotherapy. The number of regions of interest analyzed for each patient and drug condition is detailed in Supplementary Table S4. CPPT, carboplatin; PTX, paclitaxel.

Enrichment and recruitment of CD8^+^ cytolytic T cells were observed in regions of localized dazostinag exposure and were accompanied by increased expression of the chemokine CXCL9 (Fig. 4A and B). Evaluation of CosMx SMI data provided strong evidence of increased cytolytic activity by dazostinag, with a significant increase detected in a population of granulysin-expressing NK cells (designated as T_Cell_GNLY; Fig. 4C). However, this effect of dazostinag was dampened by combination with chemotherapy. Dual-positive CD86^+^/CD11c^+^ cells were also noted in regions of dazostinag exposure, suggesting its effects could include the activation of dendritic cells (Supplementary Fig. S9).

**Figure 5:**
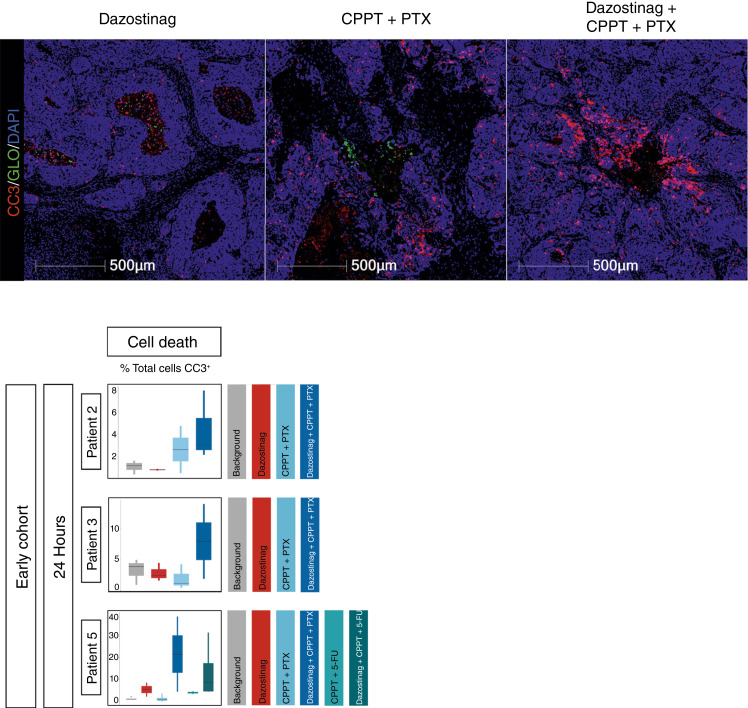
Dazostinag + CPPT + PTX induced tumor cell apoptosis at 24 hours postintratumoral injection. Staining with the apoptosis marker CC3 showed minimal change at sites with dazostinag alone, a modest increase when treated with carboplatin + paclitaxel, and significantly elevated CC3 at sites with dazostinag + carboplatin + paclitaxel. Cell nuclei, blue; CC3, red; CIVO GLO, green. The number of regions of interest analyzed for each patient and drug conditions is detailed in Supplementary Table S4. CC3, cleaved caspase-3; CPPT, carboplatin; PTX, paclitaxel.

In three patients, the IFN response was accompanied by an increase in cellular apoptosis. Dazostinag combined with carboplatin and paclitaxel induced enhanced tumor cell apoptosis versus dazostinag or chemotherapy alone, based on staining with the apoptosis marker cleaved caspase-3 in samples from three patients in the 24-hour cohort (Fig. 5). The data were consistent with those observed in the preclinical mouse model (Supplementary Fig. S4).

## Discussion

This phase 0 study employing the CIVO microdosing platform with spatial profiling and IHC assessments evaluated the immune response changes induced by the STING agonist dazostinag alone and in combination with widely employed chemotherapies within the TME of patients with HNSCC. Similar to findings from the first-of-its-kind phase 0 study, which demonstrated that the mechanism of action of the SUMOylating inhibitor subasumstat could be assessed directly in a spatially precise manner in human tumors *in situ* using CIVO technology (26), this study indicated that this platform, together with spatial profiling, is well tolerated and enables mechanistic validation and assessment of drug combinations of the novel STING agonist dazostinag in patient tumors in a minimally invasive manner. The CIVO platform, therefore, has the potential to address some of the long-standing challenges surrounding the issue of conflicting data gathered from laboratory models of cancer versus the clinical setting (24, 25, 30–35), and in doing so, may have the capability to accelerate the development of novel drug trials by discriminating between novel drug candidates that produce on-target biological responses and those that do not.

The current phase 0 study demonstrated activation of the STING pathway and concomitant activation of proinflammatory type I IFN genes after localized intratumor exposure to dazostinag. In the 24-hour cohort, dazostinag exposure resulted in active remodeling of the TME, including increased expression of proinflammatory cytokines and chemokines, recruitment of cytotoxic T cells, and M1-like proinflammatory macrophages. In further confirmation, spatial molecular profiling showed increased IFN signaling across multiple cell types and compartments throughout the TME. However, similar results were not observed in the late (≥72-hour) cohort, in which it is possible that most biomarkers had returned to baseline levels, which aligns with pharmacologic data from a dose escalation trial of dazostinag in combination with pembrolizumab in refractory solid tumors (7). The study also revealed that the combination of dazostinag with chemotherapy agents led to a similar enhancement of IFN signaling, with evidence of increased cancer cell apoptosis within 24 hours of exposure. Nevertheless, the addition of chemotherapy to dazostinag not only led to a diminished effect of dazostinag on inducing CXCL9 signaling but also resulted in the upregulation of several markers associated with a more M2-like, anti-inflammatory TME, including CD163 and CCL2, potentially dampening some of the intended effects of STING agonism. The causality behind the increase in the M2-like phenotype in samples treated with dazostinag combined with chemotherapy is unclear although it may be related to increased cell death of CXCL9-producing cells by chemotherapy.

The results presented here support findings obtained from preclinical *in vitro* and *in vivo* studies of dazostinag, demonstrating its ability to induce both innate and adaptive immune responses (1). In murine and human cell lines, dazostinag dose-dependently induced activation of the STING signaling pathway and type I IFNs. Furthermore, dazostinag was found to activate dendritic cells, NK cells, and T cells, both *in vitro* and *in vivo* in tumor-bearing syngeneic murine models. In addition to inducing dose-dependent cytokine responses *in vivo*, dazostinag increased the activation and proliferation of immune cells, such as dendritic cells and CD8^+^ T cells, within the TME and local tumor-associated lymphoid tissue.

These preclinical findings, together with data from our current study leveraging the CIVO platform and spatial profiling technology, are particularly interesting in light of the recent work of Bill and colleagues (36), proposing that tumor-associated macrophage (TAM) polarity is important in establishing a pro- or antitumor TME via the expression of *Spp1* and *Cxcl9* genes, with CXCL9^hi^ TAMs associated with better clinical outcomes versus SPP1^hi^ TAMs. Their research demonstrated that the status of CXCL9:SPP1 TAM polarity was positively associated with increased tumor infiltration of immune cells (T cells, B cells, and dendritic cells), that CXCL9:SPP1^hi^ TAM-associated pathways included IFN (α and γ) signaling in most cells, and that IFN-γ promoted CXCL9 expression in TAMs. Additional to these findings, the identification of a triple drug combination comprising a STING agonist (MSA-2), a PARP7 inhibitor, and a Toll-like receptor 7/8 agonist was shown to work in synergy to polarize TAMs toward an antitumor phenotype by upregulating CXCL9 (37). In this study, we show that CXCL9:SPP1 TAM polarity can be influenced by STING agonism, which effectively shifts the immune activation state of the TME. Specifically, our series of studies demonstrated that dazostinag was involved in the remodeling of the TME by inducing IFN signaling, activating the CXCL9-11/CXCR3 axis, inducing CXCL9 signaling and recruitment of cytotoxic T cells/activation of dendritic cells, and shifting the TME to a more proinflammatory phenotype. By extension, given that CXCL9:SPP1 TAM polarity has been shown to correlate with cancer progression and patient prognosis (36), the results shown here suggest that exposure to dazostinag may not only prime tumors for response to ICIs targeting the PD-1/PD-L1 axis but may also induce antitumor responses as a single agent by effectively shifting the immune cell composition and activity state of the TME via a primary effect on TAMs.

Further evidence for this was the colocalization of CD8 enrichment and chemokine expression seen in this study, suggesting that CXCL9 is a key driver of T-cell accumulation, a finding that has also been reported elsewhere. In a syngeneic mouse model of ovarian cancer, the number of intratumoral CD8^+^ cells was increased 2.5-fold following CXCL9 overexpression (38). Additionally, overexpression of this chemokine led to decreased peritoneal metastasis and significantly prolonged median survival in mice. Interestingly, anti–PD-L1 treatment was found to act synergistically with CXCL9 overexpression in the ovarian cancer model, resulting in a significantly prolonged time to onset of ascites and prolonged overall survival (38). Likewise, in another study, CXCL9 was upregulated in endometrial cancer, and its expression was found to be positively correlated with the infiltration of immune cells (T cells, B cells, NK cells, dendritic cells, macrophages) in the TME (39). PD-L1 was also positively related to the expression of CXCL9. Furthermore, STING-mediated IFN signaling has also been reported to increase the expression of PD-L1 (40, 41).

Together, these results suggest potential treatment strategies that combine agents involved in CXCL9 induction with anti–PD-L1 or anti–PD-1 therapy, such as the concomitant administration of STING agonists like dazostinag with pembrolizumab. Indeed, such combinations might help solve the issue of primary and secondary resistance to checkpoint inhibitors, which is associated with reduced IFN signaling and an immune-suppressive tumor phenotype, by activating type 1 IFN signaling and downstream adaptive antitumor immune mechanisms (42, 43). In this regard, a phase I/II, open-label study evaluating the safety, tolerability, pharmacokinetics, and pharmacodynamics of dazostinag alone and in combination with pembrolizumab has demonstrated early clinical responses and durable stable disease in a population of heavily pretreated patients with advanced or metastatic solid tumors, with an overall response rate of 34.5% in the HNSCC expansion cohort (NCT04420884; refs. 3, 5, 7, 44). Another phase I study of dazostinag plus pembrolizumab following radiotherapy also demonstrated clinical activity in patients with advanced or metastatic non–small cell lung cancer or HNSCC (NCT04879849; ref. 6). Pharmacodynamic analyses from these studies revealed the induction of a STING gene signature, increased cytokine production, peripheral immune cell activation, and CD8^+^ T-cell recruitment to tumors, consistent with findings from the present phase 0 study.

Our study had several limitations. First, this was a phase 0 intratumoral microdosing study, and therefore, the findings of our research are constrained to the evaluation of local pharmacodynamic responses in the TME. By the nature of the type of study conducted, the patient sample size was small; nonetheless, the number of distinct TMEs that can be evaluated per patient tumor via spatial profiling around sites of drug microdose injection largely offsets this issue. The localized microdosing of dazostinag may result in a short drug exposure time, and although this approach supports proximal mechanistic evaluations, it limits longer-duration events. This may explain the pharmacodynamic activity being observed at 24 hours but not at extended time points in our patients. In addition, due to the nature of the study, patients were enrolled in the trial as deemed eligible and without additional selection criteria, resulting in heterogeneity of samples available for the analyses (i.e., from local vs. metastatic tumors), which could have affected the immune responses observed. Heterogeneity in the TME, while acknowledged, represents the complex clinical situation and can be viewed as a strength of this approach compared with preclinical mouse tumor and *ex vivo* models. Finally, although this study provides a direct observation of localized tumor responses following exposure to investigational drugs such as dazostinag, it cannot determine whether such drugs, when delivered systemically, can reach and sufficiently penetrate the TME to induce such effects, nor can this study provide information on the safety and tolerability of systemically administered drugs. Nevertheless, despite its inherently exploratory nature, our study demonstrates the possibility of combining CIVO with spatial profiling to evaluate the immunobiology of investigative agents and, most importantly, provides clear evidence that if sufficient tumor exposure is achieved following systemic dosing, functional shifts in the state of the TME can be anticipated. Furthermore, this study provides a valuable bridge between the preclinical and clinical findings. Illustrating an ability to recapitulate preclinical drug effects in heterogeneous tumors clinically is a valuable finding, and understanding the differences between human and murine immune responses to drugs can shape the interpretation of the preclinical data. CIVO phase 0 studies provide better spatial resolution than phase I pre- and posttreatment biopsies by comparing treated and untreated areas of the same tumor at the same time point. CIVO data also provide more context for interactions between immune cell populations and tumor cells in the TME compared with posttreatment blood draws.

### Conclusions

In this phase 0 study, intratumoral microdosing via CIVO and spatial profiling of dazostinag alone and combined with chemotherapy agents was shown to stimulate innate immunity and downstream adaptive immunity, leading to an early proinflammatory response, providing evidence of an on-target mechanism of action. Moreover, the study demonstrated the utility of this technology in affirming the mechanistic effects of dazostinag on the native TME of intact human HNSCC tumors. This, therefore, represents a potentially useful approach for investigating the mechanism of action of other novel therapies and instilling confidence in the selection of potentially additive or synergistic combination agents.

## Supplementary Material

Supplementary Information Supplementary Information

Supplementary Figure S1 Figure S1. CIVO Phase 0 clinical workflow

Supplementary Figure S2 Figure S2. Dazostinag dose-response in a syngeneic mouse model.

Supplementary Figure S3 Figure S3. High-dose dazostinag (0.24 mg/mL) induced widespread cell death after 24- and 72-hours of exposure in a syngeneic mouse model.

Supplementary Figure S4 Figure S4. Cellular apoptosis with dazostinag alone, chemotherapy doublet, and dazostinag-chemotherapy triple combination in a preclinical mouse model after 24- and 72-hours of drug exposure.

Supplementary Figure S5 Figure S5. Dazostinag stimulates the STING pathway inducing interferon expression and promoting a pro-inflammatory tumor microenvironment.

Supplementary Figure S6 Figure S6. Sustained ISG15 expression at 72 hours post-intratumoral injection with dazostinag.

Supplementary Figure S7 Figure S7. Multiple cell types within the immune TME are affected by type 1 IFN response as shown by CosMx SMI including distinct macrophage subtypes of differing polarity.

Supplementary Figure S8 Figure S8. Dazostinag combinations with chemotherapy elicits an immune-suppressive response in the TME through the upregulation of CCL2.

Supplementary Figure S9 Figure S9. Dazostinag alone and combined with chemotherapy may activate dendritic cells.

Supplementary Table S1 Table S1. Primary and secondary antibodies used in the immunohistochemistry assays.

Supplementary Table S2 Table S2. Probes used for in situ hybridization.

Supplementary Table S3 Table S3. Representativeness of the study population.

Supplementary Table S4 Table S4. Number of regions of interest (ROIs) analyzed for each patient and drug condition in the boxplots shown in Figures 3, 4, and 5.

## References

[1] Carideo Cunniff E , Sato Y, Mai D, Appleman VA, Iwasaki S, Kolev V, . TAK-676: a novel stimulator of interferon genes (STING) agonist promoting durable IFN-dependent antitumor immunity in preclinical studies. Cancer Res Commun 2022;2:489–502. 36923556 10.1158/2767-9764.CRC-21-0161 PMC10010323

[2] Knelson EH , Ivanova EV, Tarannum M, Campisi M, Lizotte PH, Booker MA, . Activation of tumor-cell STING primes NK-cell therapy. Cancer Immunol Res 2022;10:947–61. 35678717 10.1158/2326-6066.CIR-22-0017 PMC9357206

[3] Falchook GS , Luke JJ, Strauss JF, Gao X, Lorusso P, Voon PJ, . A phase 1 dose-escalation study of intravenously (IV) administered TAK-676, a novel STING agonist, alone and in combination with pembrolizumab in patients (pts) with advanced or metastatic solid tumors. J Clin Oncol 2021;39(Suppl 15):TPS2670.

[4] Cooper BT , Chmura SJ, Luke JJ, Shiao SL, Basho RK, Iams WT, . TAK-676 in combination with pembrolizumab after radiation therapy in patients (pts) with advanced non–small cell lung cancer (NSCLC), triple-negative breast cancer (TNBC), or squamous-cell carcinoma of the head and neck (SCCHN): phase 1 study design. J Clin Oncol 2022;40(Suppl 16):TPS2698.

[5] Olszanski AJ , Luke JJ, LoRusso PM, Falchook GS, Bedard PL, Sanborn RE, . 1029P Dazostinag (TAK-676) alone and in combination with pembrolizumab (pembro) in patients (pts) with advanced or metastatic solid tumors: preliminary safety, PK/PD, and anti-tumor activity in a phase I dose escalation study supporting a recommended dose for expansion (RDE). Ann Oncol 2023;34(Suppl 2):S625–6.

[6] Cooper BT , Olson D, Iams WT, Page DB, Yuan Y, Gerber NK, . 778 Phase 1b study of dazostinag plus pembrolizumab after hypofractionated radiotherapy in patients with non-small-cell lung cancer, triple-negative breast cancer, or head and neck squamous-cell carcinoma. J ImmunoTher Cancer 2024;12(Suppl 2):A882.

[7] Luke JJ , Gao X, Olszanski AJ, Sanborn RE, Falchook GS, Patel S, . 996MO Dazostinag (TAK-676) alone and in combination with pembrolizumab (pembro) in patients (pts) with advanced/metastatic solid tumors: data from phase I dose escalation. Ann Oncol 2024;35(Suppl 2):S678–9.

[8] Pfister DG , Spencer S, Adelstein D, Adkins D, Anzai Y, Brizel DM, . Head and neck cancers, version 2.2020, NCCN clinical Practice guidelines in oncology. J Natl Compr Canc Netw 2020;18:873–98. 32634781 10.6004/jnccn.2020.0031

[9] Burtness B , Harrington KJ, Greil R, Soulières D, Tahara M, de Castro G Jr, . Pembrolizumab alone or with chemotherapy versus cetuximab with chemotherapy for recurrent or metastatic squamous cell carcinoma of the head and neck (KEYNOTE-048): a randomised, open-label, phase 3 study. Lancet 2019;394:1915–28. 31679945 10.1016/S0140-6736(19)32591-7

[10] Moore E , Clavijo PE, Davis R, Cash H, Van Waes C, Kim Y, . Established T cell-inflamed tumors rejected after adaptive resistance was reversed by combination STING activation and PD-1 pathway blockade. Cancer Immunol Res 2016;4:1061–71. 27821498 10.1158/2326-6066.CIR-16-0104 PMC5134907

[11] Hayman TJ , Baro M, MacNeil T, Phoomak C, Aung TN, Cui W, . STING enhances cell death through regulation of reactive oxygen species and DNA damage. Nat Commun 2021;12:2327. 33875663 10.1038/s41467-021-22572-8 PMC8055995

[12] Baird JR , Feng Z, Xiao HD, Friedman D, Cottam B, Fox BA, . STING expression and response to treatment with STING ligands in premalignant and malignant disease. PLoS One 2017;12:e0187532. 29135982 10.1371/journal.pone.0187532 PMC5685615

[13] Luo X , Donnelly CR, Gong W, Heath BR, Hao Y, Donnelly LA, . HPV16 drives cancer immune escape via NLRX1-mediated degradation of STING. J Clin Invest 2020;130:1635–52. 31874109 10.1172/JCI129497 PMC7108911

[14] Saulters EL , Kennedy PT, Carter RJ, Alsufyani A, Jones TM, Woolley JF, . Differential regulation of the STING pathway in human papillomavirus–positive and -negative head and neck cancers. Cancer Res Commun 2024;4:118–33. 38147007 10.1158/2767-9764.CRC-23-0299 PMC10793589

[15] Hanahan D , Weinberg RA. Hallmarks of cancer: the next generation. Cell 2011;144:646–74. 21376230 10.1016/j.cell.2011.02.013

[16] de Visser KE , Joyce JA. The evolving tumor microenvironment: from cancer initiation to metastatic outgrowth. Cancer Cell 2023;41:374–403. 36917948 10.1016/j.ccell.2023.02.016

[17] Xiao Y , Yu D. Tumor microenvironment as a therapeutic target in cancer. Pharmacol Ther 2021;221:107753. 33259885 10.1016/j.pharmthera.2020.107753 PMC8084948

[18] Cassetta L , Pollard JW. A timeline of tumour-associated macrophage biology. Nat Rev Cancer 2023;23:238–57. 36792751 10.1038/s41568-022-00547-1

[19] Noy R , Pollard JW. Tumor-associated macrophages: from mechanisms to therapy. Immunity 2014;41:49–61. 25035953 10.1016/j.immuni.2014.06.010 PMC4137410

[20] Garcia-Diaz A , Shin DS, Moreno BH, Saco J, Escuin-Ordinas H, Rodriguez GA, . Interferon receptor signaling pathways regulating PD-L1 and PD-L2 expression. Cell Rep 2017;19:1189–201. 28494868 10.1016/j.celrep.2017.04.031 PMC6420824

[21] Junttila MR , de Sauvage FJ. Influence of tumour micro-environment heterogeneity on therapeutic response. Nature 2013;501:346–54. 24048067 10.1038/nature12626

[22] Wong CH , Siah KW, Lo AW. Estimation of clinical trial success rates and related parameters. Biostatistics 2019;20:273–86. 29394327 10.1093/biostatistics/kxx069 PMC6409418

[23] Arrowsmith J , Miller P. Trial watch: phase II and phase III attrition rates 2011–2012. Nat Rev Drug Discov 2013;12:569. 23903212 10.1038/nrd4090

[24] Gundle KR , Deutsch GB, Goodman HJ, Pollack SM, Thompson MJ, Davis JL, . Multiplexed evaluation of microdosed antineoplastic agents in situ in the tumor microenvironment of patients with soft tissue sarcoma. Clin Cancer Res 2020;26:3958–68. 32299817 10.1158/1078-0432.CCR-20-0614

[25] Klinghoffer RA , Bahrami SB, Hatton BA, Frazier JP, Moreno-Gonzalez A, Strand AD, . A technology platform to assess multiple cancer agents simultaneously within a patient’s tumor. Sci Transl Med 2015;7:284ra58. 10.1126/scitranslmed.aaa7489 PMC4770902 25904742

[26] Derry JMJ , Burns C, Frazier JP, Beirne E, Grenley M, DuFort CC, . Trackable intratumor microdosing and spatial profiling provide early insights into activity of investigational agents in the intact tumor microenvironment. Clin Cancer Res 2023;29:3813–25. 37389981 10.1158/1078-0432.CCR-23-0827 PMC10502463

[27] US Department of Health and Human Services. Food and Drug Administration. Center for Drug Evaluation and Research . Guidance for industry, investigators, and reviewers. Exploratory IND studies. 2006. [cited 2024 Mar 14]. Available from: https://www.fda.gov/media/72325/download.

[28] Liu J , Geng X, Hou J, Wu G. New insights into M1/M2 macrophages: key modulators in cancer progression. Cancer Cell Int 2021;21:389. 34289846 10.1186/s12935-021-02089-2 PMC8296555

[29] Jin J , Lin J, Xu A, Lou J, Qian C, Li X, . CCL2: an important mediator between tumor cells and host cells in tumor microenvironment. Front Oncol 2021;11:722916. 34386431 10.3389/fonc.2021.722916 PMC8354025

[30] Hegde PS , Chen DS. Top 10 challenges in cancer immunotherapy. Immunity 2020;52:17–35. 31940268 10.1016/j.immuni.2019.12.011

[31] Hum NR , Sebastian A, Gilmore SF, He W, Martin KA, Hinckley A, . Comparative molecular analysis of cancer behavior cultured in vitro, in vivo, and ex vivo. Cancers (Basel) 2020;12:690. 32183351 10.3390/cancers12030690 PMC7140030

[32] Idrisova KF , Simon H-U, Gomzikova MO. Role of patient-derived models of cancer in translational oncology. Cancers 2022;15:139. 36612135 10.3390/cancers15010139 PMC9817860

[33] Ireson CR , Alavijeh MS, Palmer AM, Fowler ER, Jones HJ. The role of mouse tumour models in the discovery and development of anticancer drugs. Br J Cancer 2019;121:101–8. 31231121 10.1038/s41416-019-0495-5 PMC6738037

[34] Martinez-Pacheco S , O’Driscoll L. Pre-clinical in vitro models used in cancer research: results of a worldwide survey. Cancers (Basel) 2021;13:6033. 34885142 10.3390/cancers13236033 PMC8656628

[35] Sleijfer S , Lolkema M. Bridging the translational divide in oncology: in vivo testing of chemo-sensitivity. Clin Cancer Res 2020;26:3897–8. 32482905 10.1158/1078-0432.CCR-20-1452

[36] Bill R , Wirapati P, Messemaker M, Roh W, Zitti B, Duval F, . CXCL9:SPP1 macrophage polarity identifies a network of cellular programs that control human cancers. Science 2023;381:515–24. 37535729 10.1126/science.ade2292 PMC10755760

[37] Enbergs N , Halabi EA, Goubet AG, Schleyer K, Fredrich IR, Kohler RH, . Pharmacological polarization of tumor-associated macrophages toward a CXCL9 antitumor phenotype. Adv Sci (Weinh) 2024;11:e2309026. 38342608 10.1002/advs.202309026 PMC11022742

[38] Seitz S , Dreyer TF, Stange C, Steiger K, Bräuer R, Scheutz L, . CXCL9 inhibits tumour growth and drives anti-PD-L1 therapy in ovarian cancer. Br J Cancer 2022;126:1470–80. 35314795 10.1038/s41416-022-01763-0 PMC9090786

[39] Xue S , Su X-M, Ke L-N, Huang Y-G. CXCL9 correlates with antitumor immunity and is predictive of a favorable prognosis in uterine corpus endometrial carcinoma. Front Oncol 2023;13:1077780. 36845675 10.3389/fonc.2023.1077780 PMC9945585

[40] Cha J-H , Chan L-C, Li C-W, Hsu JL, Hung M-C. Mechanisms controlling PD-L1 expression in cancer. Mol Cell 2019;76:359–70. 31668929 10.1016/j.molcel.2019.09.030 PMC6981282

[41] Grabosch S , Bulatovic M, Zeng F, Ma T, Zhang L, Ross M, . Cisplatin-induced immune modulation in ovarian cancer mouse models with distinct inflammation profiles. Oncogene 2019;38:2380–93. 30518877 10.1038/s41388-018-0581-9 PMC6440870

[42] Nowicki TS , Hu-Lieskovan S, Ribas A. Mechanisms of resistance to PD-1 and PD-L1 blockade. Cancer J 2018;24:47–53. 29360728 10.1097/PPO.0000000000000303 PMC5785093

[43] Walsh RJ , Soo RA. Resistance to immune checkpoint inhibitors in non-small cell lung cancer: biomarkers and therapeutic strategies. Ther Adv Med Oncol 2020;12:1758835920937902. 32670423 10.1177/1758835920937902 PMC7339077

[44] Fayette J , Popovtzer A, Forster MD, Adkins D, Guo Y, Lefebvre G, . Dose expansion data from iintune-1, a phase 1/2 study of the STING agonist dazostinag plus pembrolizumab as first-line (1L), in patients with recurrent/metastatic squamous cell carcinoma of the head and neck (RM-SCCHN). J Clin Oncol 2025(Suppl 16):6020.

[45] DeTora LM , Toroser D, Sykes A, Vanderlinden C, Plunkett FJ, Lane T, . Good publication practice (GPP) guidelines for company-sponsored biomedical research: 2022 update. Ann Intern Med 2022;175:1298–304. 36037471 10.7326/M22-1460

