# Supplementary material for: In Situ Proinflammatory Effects of Dazostinag Alone or with Chemotherapy on the Tumor Microenvironment of Patients with Head and Neck Squamous Cell Carcinoma
Source: Cancer Res Commun. 2025 Jul 30;5(7):1243–55. doi: 10.1158/2767-9764.CRC-25-0314 (PMC12308172; doi:10.1158/2767-9764.CRC-25-0314)
Supplement: Supplementary Information [file crc-25-0314_supplementary_information_suppsi.docx]

## Supplementary Information

*In Situ* Pro-Inflammatory Effects of Dazostinag Alone or With Chemotherapy Upon the Tumor Microenvironment of Patients With Head and Neck Squamous Cell Carcinoma

Richard C. Gregory, Neil Lineberry, Alex Parent, Karthik Rajasekaran, Thomas J. Ow, Cherie-Ann Nathan, Beryl A. Hatton, Wendy Jenkins, Marc Grenley, Connor Burns, Angela Merrell, Jason P. Frazier, Jonathan M.J. Derry, Emily Beirne, and Richard A. Klinghoffer

### Eligibility Criteria

Inclusion Criteria

1. Ability and willingness to comply with the study’s visit and assessment schedule.
2. Male or female ≥18 years of age at visit 1 (screening).
3. Pathologic diagnosis of head and neck squamous cell carcinoma (HNSCC).
4. Ability and willingness to provide written informed consent. Voluntary written consent must be given before performance of any study-related procedure not part of standard medical care, with the understanding that consent may be withdrawn by the patient at any time without prejudice to future medical care.
5. At least one lesion (primary tumor, recurrent tumor, or effaced metastatic lymph node) ≥2 cm in the shortest diameter that is surface accessible for comparative *in vivo* oncology (CIVO) injection that may be guided by ultrasound if appropriate and for which there is a planned surgical intervention. Treatment plan may include adjuvant radiation or chemotherapy and patients should have no medical contraindication to surgery.
6. Eastern Cooperative Oncology Group performance status of 0–2.
7. Female patients who:
   1. Are postmenopausal for at least one year before the screening visit, OR
   2. Are surgically sterile, OR
   3. Are of childbearing potential who agree to practice a highly effective method of contraception and one additional effective (barrier) method at the same time (see examples below) from the time of signing the informed consent form (ICF) through four months after the tumor injection procedure OR agree to completely abstain from heterosexual intercourse.

Highly effective methods:

- - - Intra-uterine devices.
    - Hormonal (birth control pills/oral contraceptives, injectable contraceptives, contraceptive patches, or contraceptive implants).

Other effective methods (barrier methods):

- Latex condom.
- Diaphragm with spermicide; cervical cap; sponge.
- Agree to refrain from donating ova during study participation and up to four months after the tumor injection procedure.

1. Male patients, even if surgically sterile (i.e., status post-vasectomy), who:
   1. Agree to practice effective barrier contraception from the time of signing the ICF through four months after the tumor injection procedure OR agree to
   2. Completely abstain from heterosexual intercourse.
   3. Agree to refrain from donating sperm during study participation and up to four months after the tumor injection procedure.

Exclusion criteria

1. Tumors or effaced nodes that are anticipated by the investigator to lack a sufficient volume of viable tumor tissue (based on available pre-operative imaging, pre-injection ultrasound imaging, or pathology reports) for CIVO injection due to size, location, necrosis, cysts, excessive stroma, or fibrosis.
2. Patients who have received neoadjuvant therapy associated with the surgical intervention described in inclusion criterion #5.
3. Tumors near or involving critical structures for which, in the opinion of the treating clinician, injection would pose undue risk to the patient.
4. Female patients who are:
   1. Both lactating and breastfeeding, OR
   2. Have a positive β-human chorionic gonadotropin pregnancy test at screening verified by the investigator.
5. Any uncontrolled intercurrent illness, condition, serious medical or psychiatric illness, or circumstance that, in the opinion of the investigator, could interfere with adherence to the study’s procedures or requirements, or otherwise compromise the study’s

objectives.

1. Patients with a history of concurrent second cancers requiring active, ongoing systemic treatment.
2. Patients with active autoimmune diseases requiring treatment or a known history of uncontrolled autoimmune disorders.
3. Patients with known HIV/AIDS with uncontrolled viral load and CD4 less than 200, a known history of other relevant congenital or acquired immunodeficiencies, or known chronic hepatitis B/C.
4. Patients that have received a live vaccine within 4 weeks of the baseline/screening visit.
5. Use of any of the following ≤2 weeks prior to CIVO injection:
   1. Chronic systemic immunosuppressive therapy or corticosteroids. Intranasal, inhaled, topical, or local corticosteroid injections (e.g., intra-articular injection), or steroids as premedication for hypersensitivity reactions (e.g., computed tomography [CT] scan premedication) are exceptions to this criterion.
   2. Biological response modifiers for treatment of active autoimmune disease.
   3. Hematopoietic growth factors
6. Patients with prior treatment with other stimulator of interferon genes (STING) agonist/antagonist and toll-like receptor (TLR) agonists, or cell therapies within 2 months of the baseline/screening visit.
7. Patients receiving concurrent systemic therapy (e.g., chemotherapy, targeted agent, or immunotherapy, etc.) or radiation therapy 4 weeks prior to screening through the planned surgical intervention.

### Dose-finding study in syngeneic mouse model

The methodology for preclinical CIVO experiments in mice have been described previously (1). Initial CIVO intratumoral microdosing studies were conducted in a preclinical syngeneic mouse model derived from the YUMM 1.7 melanoma cell line to determine the optimal dose and timepoint for clinical investigation. Timepoints of 4-and 24-hours were chosen to investigate rapid induction of an interferon response, and 72-hours to determine if there was a sustained immune response. Low, intermediate, and high doses of dazostinag were selected at 0.0125 mg/mL, 0.05 mg/mL, and 0.24 mg/mL, respectively.

At the 4-hour timepoint, all tested concentrations of dazostinag induced expression of the immune activating cytokine CXCL10 and pIRF3 in a dose-dependent manner, with an increase in the cellular apoptosis marker Cleaved Caspase 3 (CC3) observed at the highest concentration of dazostinag, i.e., 0.24 mg/mL (**Supplementary Fig. S1**). As this dose led to significant levels of cell death, potentially masking any immune-specific response and the ability to observe combination effects with chemotherapy, dazostinag 0.05 mg/mL was selected as the optimal dose for use in future phase 0 studies (**Supplementary Fig. S2**). Further studies with drug combinations showed a synergistic response with dazostinag in inducing an apoptotic response relative to chemotherapy alone (**Supplementary Fig. S3**).

### Analysis of CIVO microdosed tumors with spatial profiling

Formalin-fixed, paraffin-embedded (FFPE) tissue sections were further analyzed with the GeoMx digital spatial profiler (DSP) and CosMx spatial molecular imager (CosMx SMI) yielding spatial multiomics data at subcellular resolution. These analyses enabled insight into activated pathways, cell-cell interactions, cell atlasing, and cellular phenotyping of the tumor microenvironment (TME) in response to dazostinag exposure.

**GeoMx DSP**

To confirm the ability of dazostinag to remodel the TME through interferon signaling, spatial transcriptomic analysis with NanoString GeoMx DSP platform (NanoString Cancer Transcriptome Atlas probe set, 1864 gene targets) was performed on one patient sample at 24 hours. Unmasked, panCK-positive (tumor), and panCK-negative (TME) areas were collected and analysis was performed on each group. Differential gene expression analysis was performed to identify genes showing at least a 1.5-fold up- or down-regulation (adjusted p-value <0.01) in regions exposed to dazostinag, chemotherapy, or the combination compared with control regions. Subsequent gene enrichment analysis was performed with the BROAD Molecular Signatures Database (MSigDB).

**CosMx SMI**

To examine the effects of dazostinag in the TME at single-cell resolution, spatial multiomics with the CosMx SMI was performed on one patient sample. This platform enables rapid *in situ* spatial multiomics of up to 1,000 RNA and 100 protein analytes by performing sequential cycles of nucleic acid hybridization of fluorescent barcodes on FFPE tissues (2). Pairing this technology with the CIVO platform yields spatially resolved information on how drug exposure affects cellular phenotype and interactions, as well as changes in gene expression, thus enabling improved guidance for drug design and clinical decisions.

### Supplementary references

1. Dey J, Kerwin WS, Grenley MO, Casalini JR, Tretyak I, Ditzler SH, *et al*. Platform for rapid, quantitative assessment of multiple drug combinations simultaneously in solid tumors in vivo. *PLoS One* 2016;**11**(6): e0158617 doi: 10.1371/journal.pone.0158617.
2. He S, Bhatt R, Brown C, Brown EA, Buhr DL, Chantranuvatana K, *et al*. High-plex imaging of RNA and proteins at subcellular resolution in fixed tissue by spatial molecular imaging. *Nature Biotechnology.* 2022;**40**(12):1794-806 doi: 10.1038/s41587-022-01483-z.
