# Supplementary material for: In Situ Proinflammatory Effects of Dazostinag Alone or with Chemotherapy on the Tumor Microenvironment of Patients with Head and Neck Squamous Cell Carcinoma
Source: Cancer Res Commun. 2025 Jul 30;5(7):1243–55. doi: 10.1158/2767-9764.CRC-25-0314 (PMC12308172; doi:10.1158/2767-9764.CRC-25-0314)
Supplement: Supplementary Figure S1 — Figure S1. CIVO Phase 0 clinical workflow [file crc-25-0314_supplementary_figure_s1_suppsf1.docx]

**Supplementary Figure S1.** CIVO Phase 0 clinical workflow


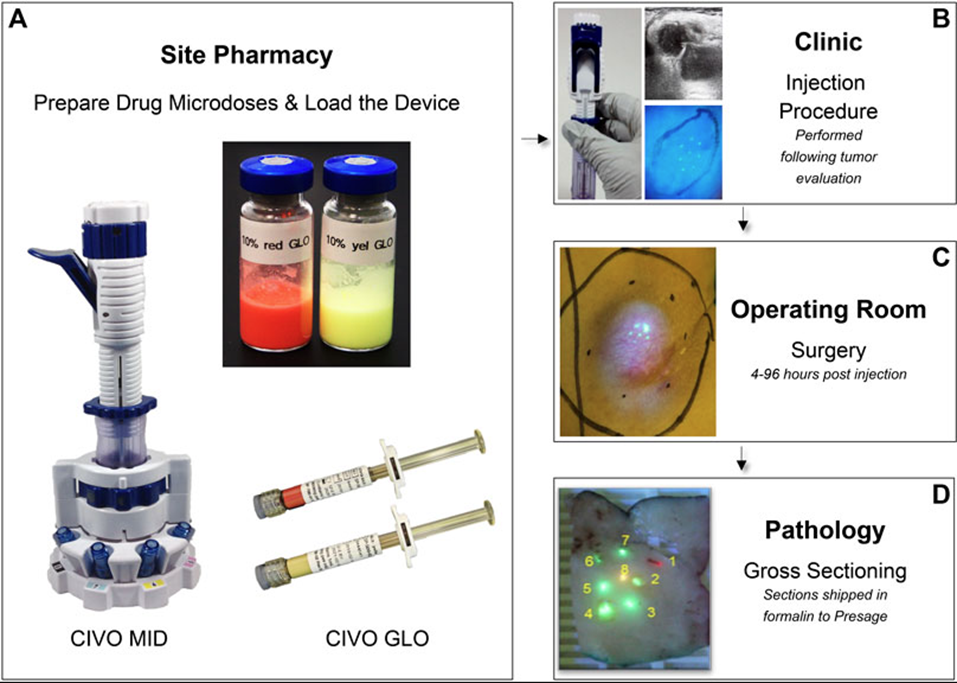


Drugs are mixed with CIVO GLO (Yellow or Red) under aseptic conditions in the site pharmacy, per study-speciﬁc instructions in vials and loaded into the handheld microdose injector (MID) transfer vessel using luer-lock syringes (A). Once loaded, the injector is transferred from the pharmacist to the investigator performing the injection. Immediately prior to an injection, ultrasonography is used to record the dimensions of the mass, evaluate the internal architecture of the tumor, identify the optimal injection placement, and customize the MID to the patient’s tumor (adjusting the depth of needle insertion and length of deposited column). During an injection, needles are carefully inserted into the tumor, and then, upon lever actuation, are retracted slowly within the tumor tissue to simultaneously deposit distinct and trackable drug columns containing minute volumes (up to 8.3 µL) of each drug, drug combination, or control. This is performed as an outpatient procedure (B). Following injection, 4–96 h later, the patient returns to the site for the scheduled surgical resection of the injected tumor, per the patient’s standard of care plan (C). The excised tumor is then transferred to pathology, where the injected portion of the tumor sample is identiﬁed, using custom blue light and yellow ﬁlter lens, then cut out, sectioned transverse to the injection columns in ~4 mm sections and placed into 10% buffered formalin containing 0.92 mg/mL sodium orthovanadate, 1.5 mg/mL sodium glycerophosphate, 1 mg/mL sodium ﬂuoride, and 2.2 mg/mL sodium pyrophosphate and shipped at room temperature (D).

Figure reused from Gundle KR, et al. (2024) Early, precise, and safe clinical evaluation of the pharmacodynamic effects of novel agents in the intact human tumor microenvironment. Frontiers Pharmacol. 15:1367581. doi: 10.3389/fphar.2024.1367581.

**Copyright** © 2024 Gundle, Rajasekaran, Houlton, Deutsch, Ow, Maki, Pang, Nathan, Clayburgh, Newman, Brinkmann, Wagner, Pollack, Thompson, Li, Mehta, Schiff, Wenig, Swiecicki, Tang, Davis, van Zante, Bertout, Jenkins, Turner, Grenley, Burns, Frazier, Merrell, Sottero, Derry, Gillespie, Mills and Klinghoffer. This is an open-access article distributed under the terms of the [Creative Commons Attribution License (CC BY).](http://creativecommons.org/licenses/by/4.0/) The use, distribution or reproduction in other forums is permitted, provided the original author(s) and the copyright owner(s) are credited and that the original publication in this journal is cited, in accordance with accepted academic practice. No use, distribution or reproduction is permitted which does not comply with these terms.
