# Supplementary material for: In Situ Proinflammatory Effects of Dazostinag Alone or with Chemotherapy on the Tumor Microenvironment of Patients with Head and Neck Squamous Cell Carcinoma
Source: Cancer Res Commun. 2025 Jul 30;5(7):1243–55. doi: 10.1158/2767-9764.CRC-25-0314 (PMC12308172; doi:10.1158/2767-9764.CRC-25-0314)
Supplement: Supplementary Figure S2 — Figure S2. Dazostinag dose-response in a syngeneic mouse model. [file crc-25-0314_supplementary_figure_s2_suppsf2.docx]

### Supplementary Figure S2. Dazostinag dose-response in a syngeneic mouse model.


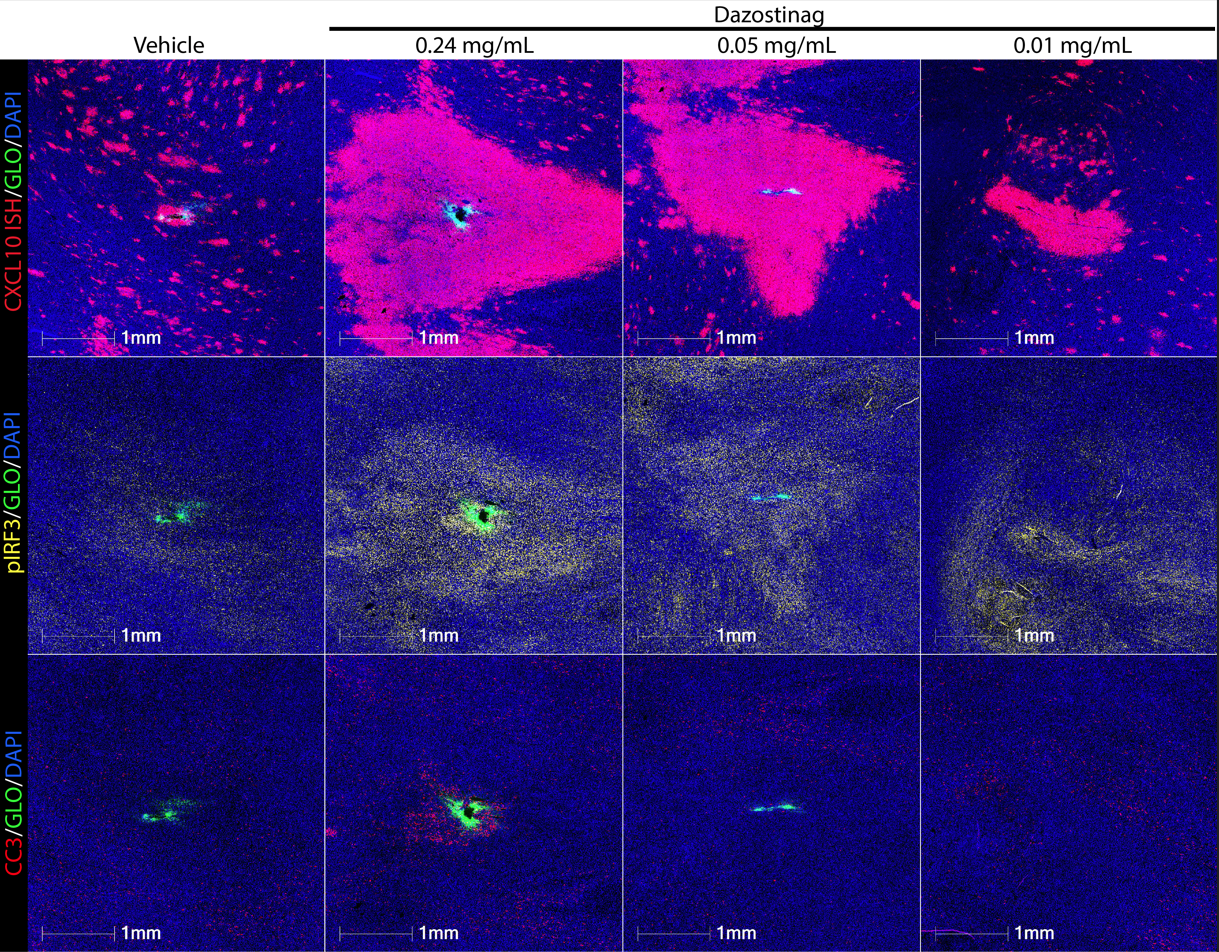


Abbreviations: CC3, Cleaved Caspase 3; CIVO, comparative *in vivo* oncology; pIRF3, phospho-interferon regulatory factor 3.

Dazostinag induced dose-dependent interferon pathway activation after 4 hours as evidenced by upregulation of CXCL10 (top; red) and pIRF3 (middle; yellow) and an increase in the apoptosis marker CC3 (bottom, red) observed at 0.24 mg/mL. Cell nuclei are depicted in blue and CIVO GLO in green.
