# Supplementary material for: In Situ Proinflammatory Effects of Dazostinag Alone or with Chemotherapy on the Tumor Microenvironment of Patients with Head and Neck Squamous Cell Carcinoma
Source: Cancer Res Commun. 2025 Jul 30;5(7):1243–55. doi: 10.1158/2767-9764.CRC-25-0314 (PMC12308172; doi:10.1158/2767-9764.CRC-25-0314)
Supplement: Supplementary Figure S3 — Figure S3. High-dose dazostinag (0.24 mg/mL) induced widespread cell death after 24- and 72-hours of exposure in a syngeneic mouse model. [file crc-25-0314_supplementary_figure_s3_suppsf3.docx]

### Supplementary Figure S3. High-dose dazostinag (0.24 mg/mL) induced widespread cell death after 24- and 72-hours of exposure in a syngeneic mouse model.


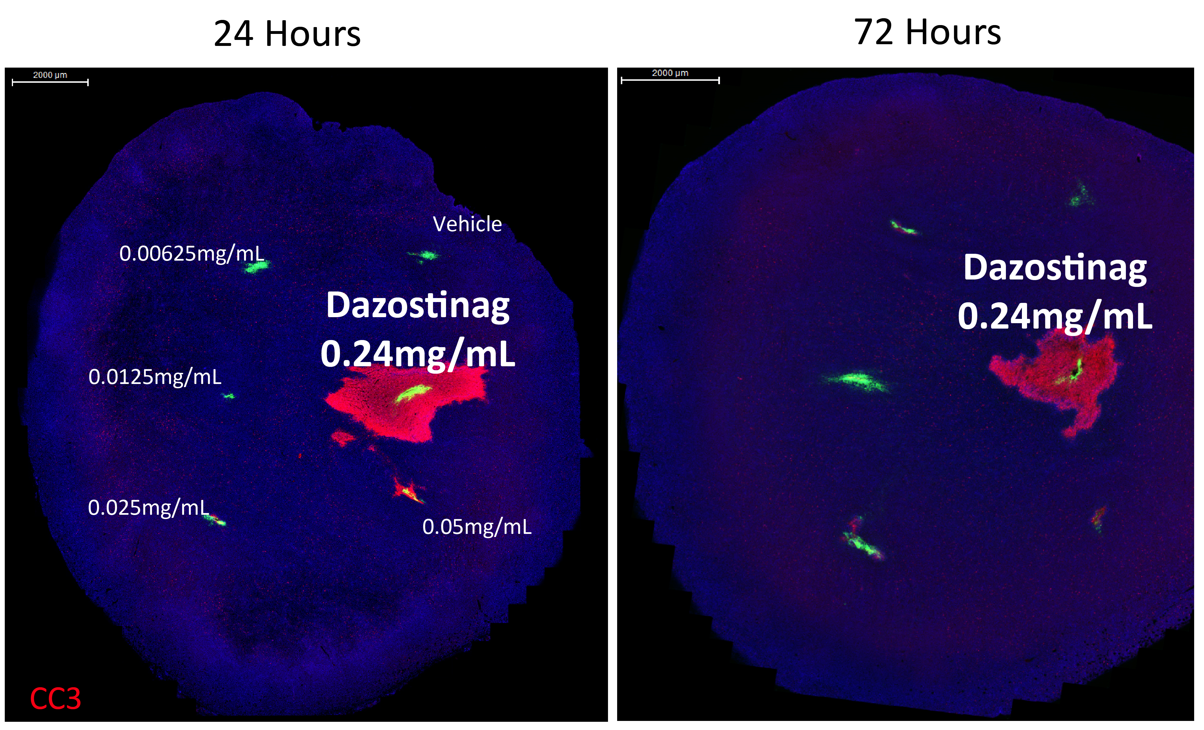


Abbreviations: CC3, Cleaved Caspase 3; CIVO, comparative *in vivo* oncology.

Tumors in a syngeneic mouse model were exposed to multiple doses of dazostinag and assayed for cellular apoptosis by CC3. Cell nuclei are depicted in blue, CC3 in red, and CIVO GLO in green.
