# Supplementary material for: In Situ Proinflammatory Effects of Dazostinag Alone or with Chemotherapy on the Tumor Microenvironment of Patients with Head and Neck Squamous Cell Carcinoma
Source: Cancer Res Commun. 2025 Jul 30;5(7):1243–55. doi: 10.1158/2767-9764.CRC-25-0314 (PMC12308172; doi:10.1158/2767-9764.CRC-25-0314)
Supplement: Supplementary Figure S4 — Figure S4. Cellular apoptosis with dazostinag alone, chemotherapy doublet, and dazostinag-chemotherapy triple combination in a preclinical mouse model after 24- and 72-hours of drug exposure. [file crc-25-0314_supplementary_figure_s4_suppsf4.docx]

### Supplementary Figure S4. Cellular apoptosis with dazostinag alone, chemotherapy doublet, and dazostinag-chemotherapy triple combination in a preclinical mouse model after 24- and 72-hours of drug exposure.


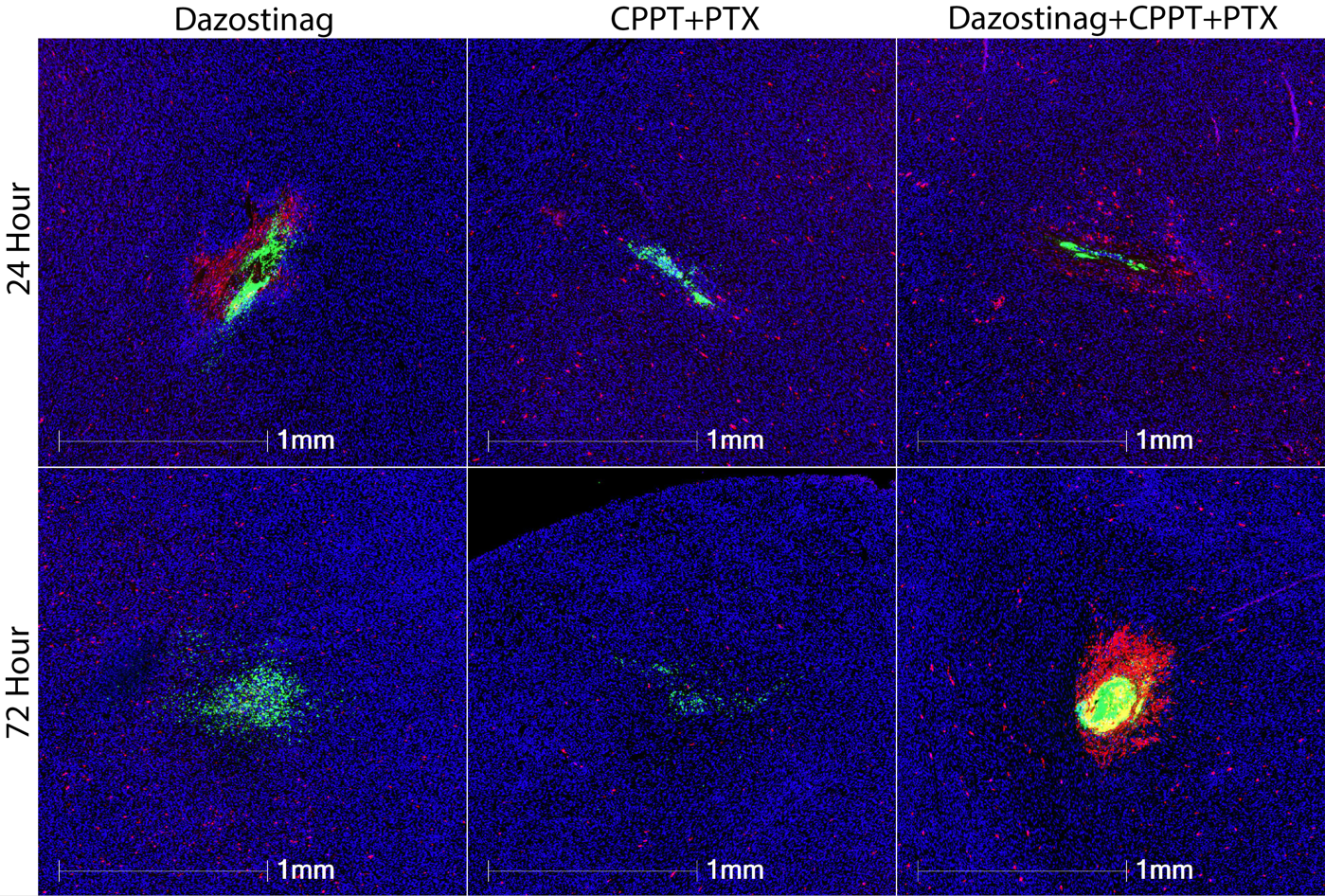


Abbreviations: CC3, Cleaved Caspase 3; CIVO, comparative *in vivo* oncology; CPPT, carboplatin; PTX, paclitaxel.

Tumors in a syngeneic mouse model showed an apoptotic response when treated with dazostinag and chemotherapy combinations at 24 and 72 hours. CC3 is depicted in red, DAPI in blue, and CIVO GLO in green.
