# Supplementary material for: In Situ Proinflammatory Effects of Dazostinag Alone or with Chemotherapy on the Tumor Microenvironment of Patients with Head and Neck Squamous Cell Carcinoma
Source: Cancer Res Commun. 2025 Jul 30;5(7):1243–55. doi: 10.1158/2767-9764.CRC-25-0314 (PMC12308172; doi:10.1158/2767-9764.CRC-25-0314)
Supplement: Supplementary Figure S5 — Figure S5. Dazostinag stimulates the STING pathway inducing interferon expression and promoting a pro-inflammatory tumor microenvironment. [file crc-25-0314_supplementary_figure_s5_suppsf5.docx]

### Supplementary Figure S5. Dazostinag stimulates the STING pathway inducing interferon expression and promoting a pro-inflammatory tumor microenvironment.
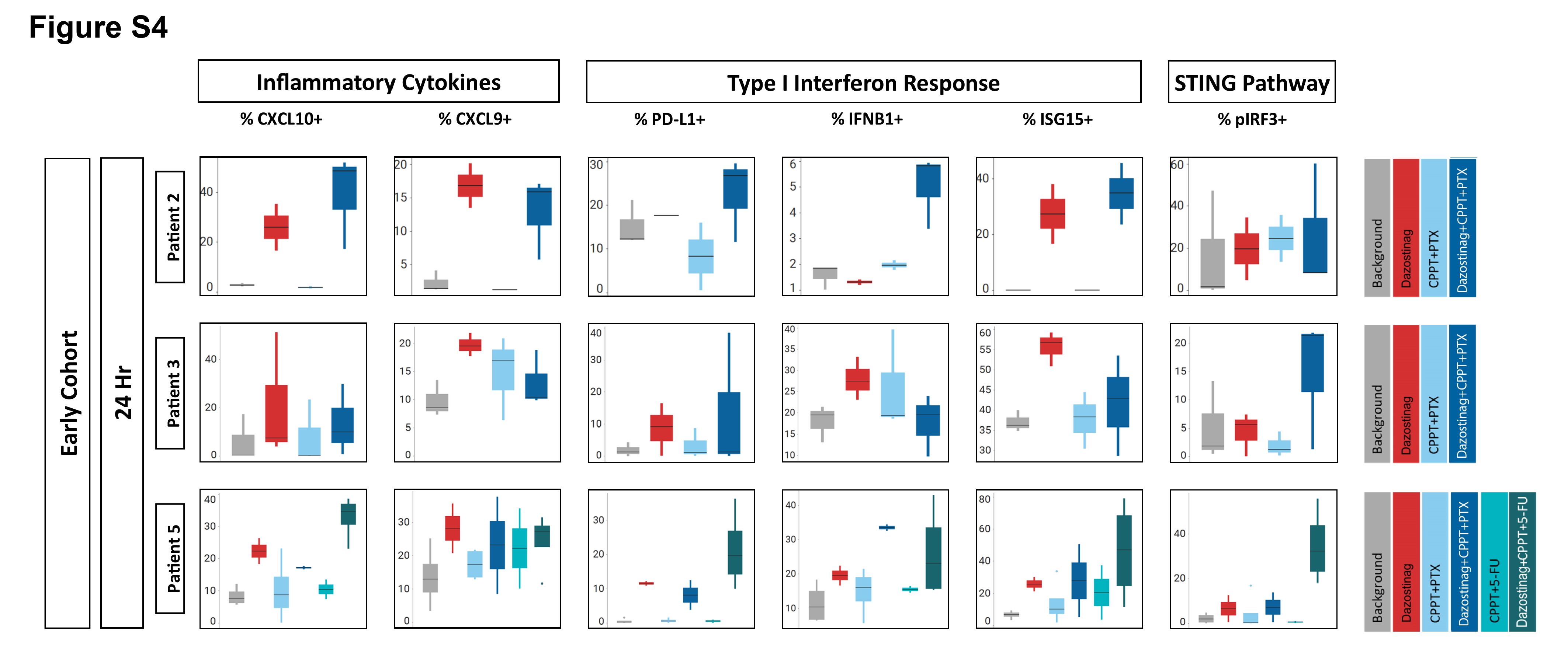


Abbreviations: 5-FU, 5-fluorouracil; CPPT, carboplatin; IFNB1, interferon beta 1; ISG15, interferon-stimulated gene 15; PD-L1, programmed cell death-ligand 1; pIRF3, phospho-interferon regulatory factor 3; PTX, paclitaxel; STING, stimulator of interferon genes.

Data shown are for quantification at 24 hours.
