# Supplementary material for: In Situ Proinflammatory Effects of Dazostinag Alone or with Chemotherapy on the Tumor Microenvironment of Patients with Head and Neck Squamous Cell Carcinoma
Source: Cancer Res Commun. 2025 Jul 30;5(7):1243–55. doi: 10.1158/2767-9764.CRC-25-0314 (PMC12308172; doi:10.1158/2767-9764.CRC-25-0314)
Supplement: Supplementary Figure S6 — Figure S6. Sustained ISG15 expression at 72 hours post-intratumoral injection with dazostinag. [file crc-25-0314_supplementary_figure_s6_suppsf6.docx]

### Supplementary Figure S6. Sustained ISG15 expression at 72 hours post-intratumoral injection with dazostinag.

1. **(B)**


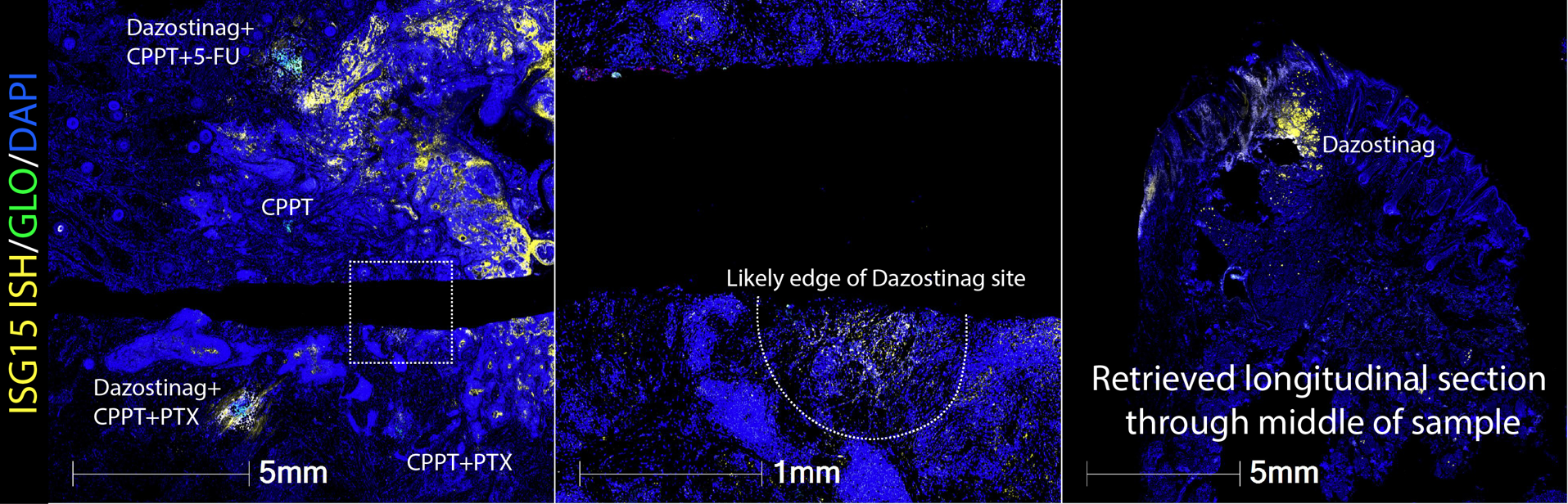


Abbreviations: 5-FU, 5-fluorouracil; CPPT, carboplatin; ISG15, interferon-stimulated gene 15; ISH, *in* situ hybridization; PTX, paclitaxel.

ISG15 (yellow) levels remained elevated after 72 hours of dazostinag exposure in one patient on dazostinag + chemotherapy combination sites (A) and as monotherapy (B). Cell nuclei are stained with DAPI (blue) and CIVO GLO (green).
