# Supplementary material for: In Situ Proinflammatory Effects of Dazostinag Alone or with Chemotherapy on the Tumor Microenvironment of Patients with Head and Neck Squamous Cell Carcinoma
Source: Cancer Res Commun. 2025 Jul 30;5(7):1243–55. doi: 10.1158/2767-9764.CRC-25-0314 (PMC12308172; doi:10.1158/2767-9764.CRC-25-0314)
Supplement: Supplementary Figure S7 — Figure S7. Multiple cell types within the immune TME are affected by type 1 IFN response as shown by CosMx SMI including distinct macrophage subtypes of differing polarity. [file crc-25-0314_supplementary_figure_s7_suppsf7.docx]

### Supplementary Figure S7. Multiple cell types within the immune TME are impacted by type 1 IFN response as shown by CosMx SMI including distinct macrophage subtypes of differing polarity. Top panels: multiple drug-exposed and untreated regions throughout the tissue section at 19 independent fields of view; bottom panels: after filtering, 64,525 cells were identified and retained for processing; 18 distinct cell types associated with the TME were identified.


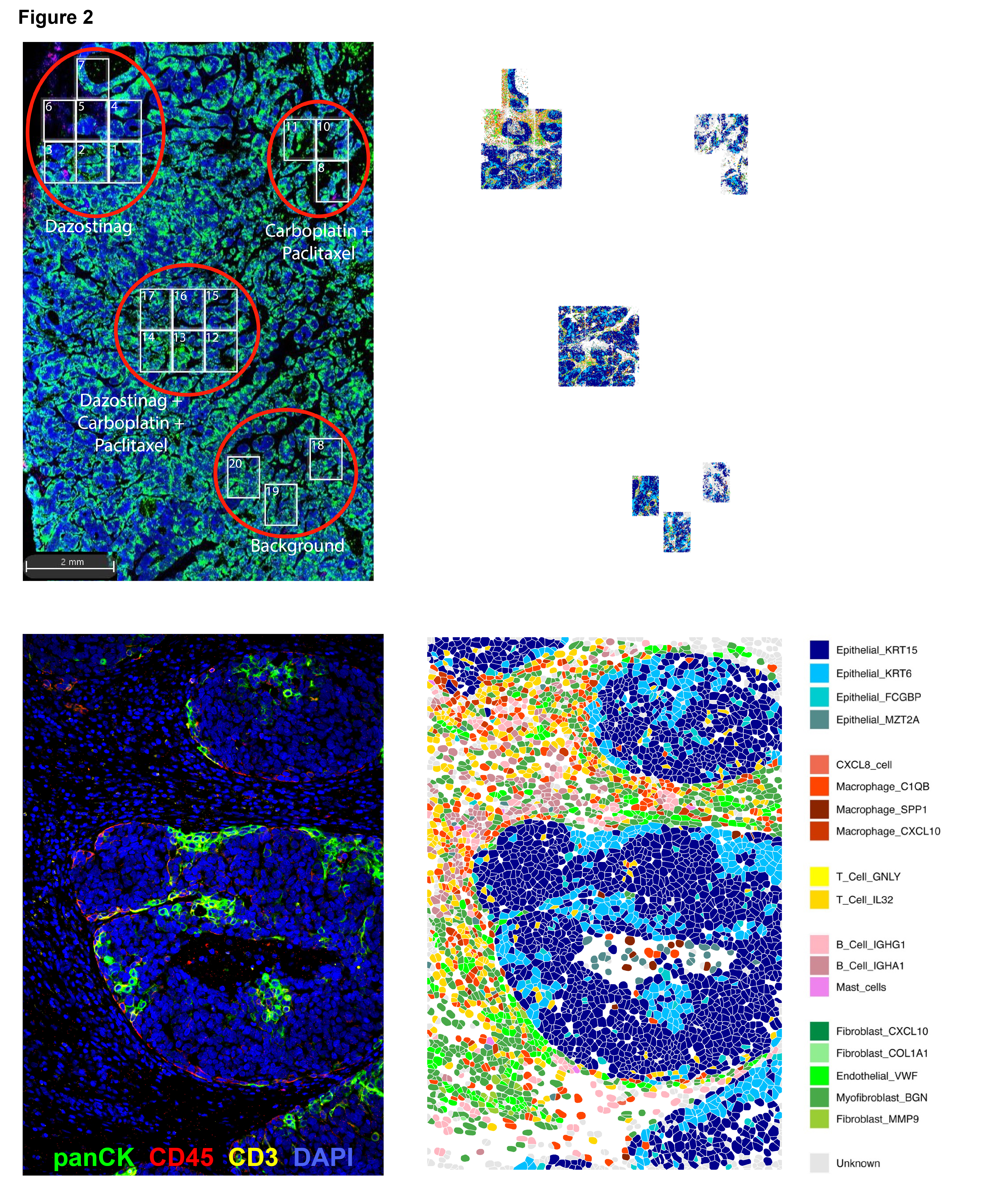


Abbreviations: IFN, interferon; panCK, pan-cytokeratin; SMI, spatial molecular imaging; TME, tumor microenvironment.

Regions of interest used for CosMx spatial molecular analysis and representative image of a reconstructed region showing the spatially resolved distribution of cell types identified throughout the TME. Cells are colored according to gene expression profiles identifying distinct cell populations. Epithelial cells are colored in blue (Epithelial_MZT2A, Epithelial_KRT6, Epithelial FCGBP, Epithelial_KRT15), macrophages in red (CXCL8_cell, Macrophage_C1QB, Macrophage_CXCL10, Macrophage_SPP1), T-cells in yellow (T_Cell_GNLY, T_Cell_IL32), B cells and mast cells in pink (B_Cell_IGHA1, B_Cell_IGHG1, Mast_cells), and fibroblasts in green (Fibroblast_COL1A1, Fibroblast_CXCL10, Fibroblast_MMP9, Myofibroblast_BGN, Endothelial_VWF).
