# Supplementary material for: In Situ Proinflammatory Effects of Dazostinag Alone or with Chemotherapy on the Tumor Microenvironment of Patients with Head and Neck Squamous Cell Carcinoma
Source: Cancer Res Commun. 2025 Jul 30;5(7):1243–55. doi: 10.1158/2767-9764.CRC-25-0314 (PMC12308172; doi:10.1158/2767-9764.CRC-25-0314)
Supplement: Supplementary Figure S8 — Figure S8. Dazostinag combinations with chemotherapy elicits an immune-suppressive response in the TME through the upregulation of CCL2. [file crc-25-0314_supplementary_figure_s8_suppsf8.docx]

### Supplementary Figure S8. Dazostinag combinations with chemotherapy elicits an immune-suppressive response in the TME through the upregulation of CCL2.


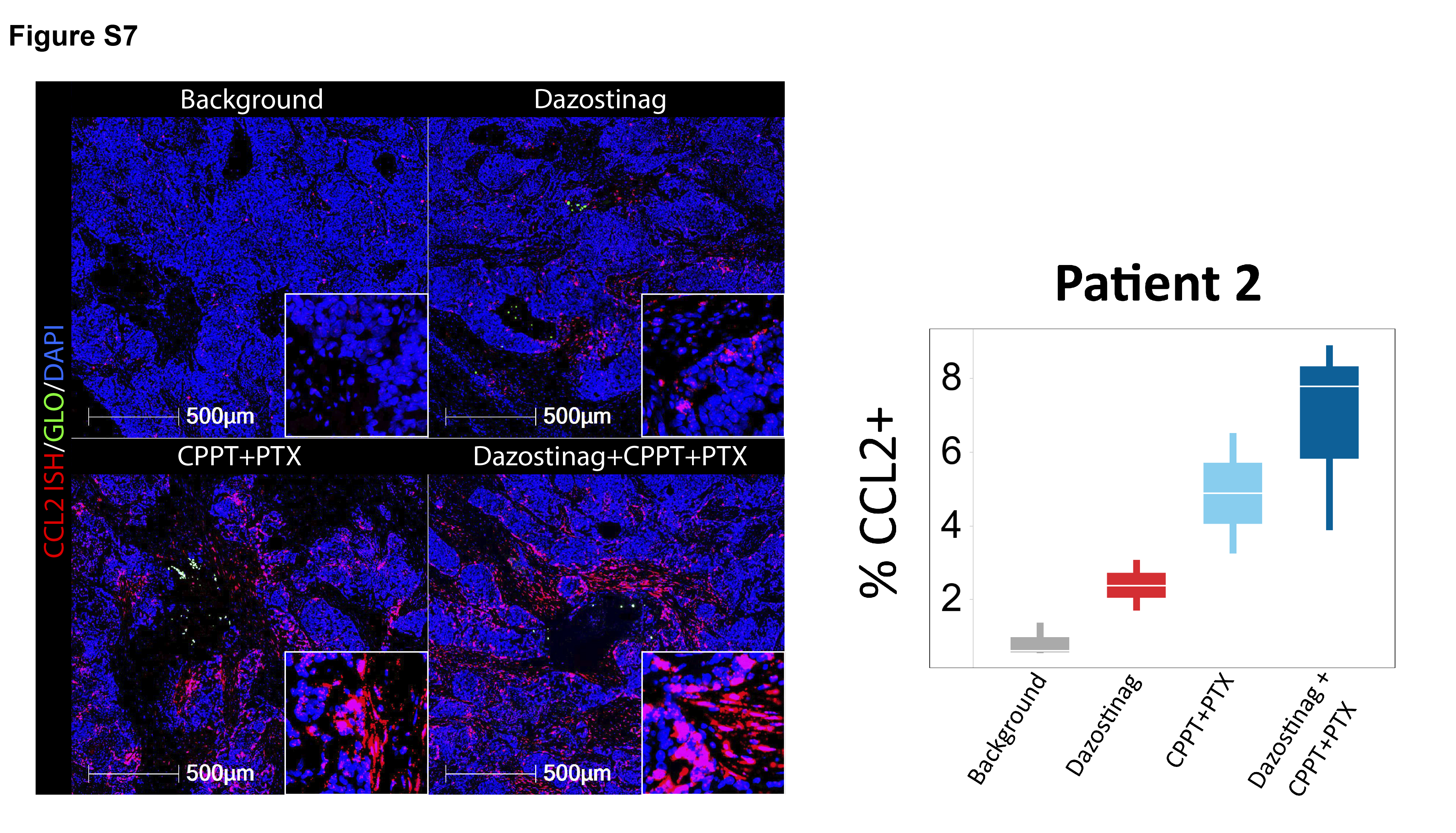


Abbreviations: CPPT, carboplatin; ISH, *in situ* hybridization*;* PTX, paclitaxel; TME, tumor microenvironment.

In situ hybridization with a probe for CCL2 (red) revealed increased expression when exposed to chemotherapy or in the triple combination relative to dazostinag alone, resulting in a more anti-inflammatory TME.
