# Supplementary material for: In Situ Proinflammatory Effects of Dazostinag Alone or with Chemotherapy on the Tumor Microenvironment of Patients with Head and Neck Squamous Cell Carcinoma
Source: Cancer Res Commun. 2025 Jul 30;5(7):1243–55. doi: 10.1158/2767-9764.CRC-25-0314 (PMC12308172; doi:10.1158/2767-9764.CRC-25-0314)
Supplement: Supplementary Figure S9 — Figure S9. Dazostinag alone and combined with chemotherapy may activate dendritic cells. [file crc-25-0314_supplementary_figure_s9_suppsf9.docx]

### Supplementary Figure S9. Dazostinag alone and combined with chemotherapy may activate dendritic cells.


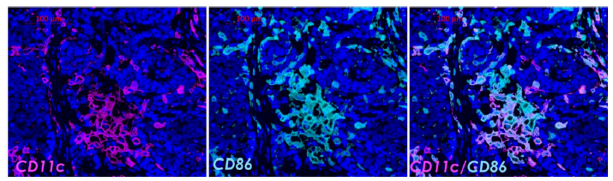


Dendritic cell activation increased in areas of dazostinag exposure shown by dual expression of CD11c (purple) and CD86 (cyan). Blue indicates DAPI.
