# Supplementary material for: In Situ Proinflammatory Effects of Dazostinag Alone or with Chemotherapy on the Tumor Microenvironment of Patients with Head and Neck Squamous Cell Carcinoma
Source: Cancer Res Commun. 2025 Jul 30;5(7):1243–55. doi: 10.1158/2767-9764.CRC-25-0314 (PMC12308172; doi:10.1158/2767-9764.CRC-25-0314)
Supplement: Supplementary Table S1 — Table S1. Primary and secondary antibodies used in the immunohistochemistry assays. [file crc-25-0314_supplementary_table_s1_suppst1.docx]

### Supplementary Table S1. Primary and secondary antibodies used in the immunohistochemistry assays.

| **Antibody** | **Source** | **Identifier** | **RRID** | **Clone** | **Dilution** | **Host species** |
| --- | --- | --- | --- | --- | --- | --- |
| **Primary antibodies** | | | | | | |
| Cleaved Caspase-3 | Cell Signaling | #9661 | AB_2341188 | Asp175 | 1:15,000 | Rabbit |
| CD163 | Novus Biologicals | #NB110-59935 | AB_892323 | 10D6 | 1:1,000 | Mouse |
| CD56 | Cell Marque | #156R-94 | AB_3082973 | MRQ-42 | 1:1,000 | Rabbit |
| CD68 | Dako | #M081401-2 | AB_2750584 | KP1 | 1:1,000 | Mouse |
| CD8 | Abcam | #ab17147 | AB_443686 | 144B | 1:1,000 | Mouse |
| CD86 | Cell Signaling | #91882 | AB_2797422 | E2G8P | 1:2,500 | Rabbit |
| Granzyme B | Abcam | #ab134933 | AB_2889221 | EPR8260 | 1:5,000 | Rabbit |
| Mac387 | Dako | #M0747 | AB_3675660 | MAC387 | 1:1,000 | Mouse |
| P16 | Biocare Medical | #3231 | AB_2942034 | BC42 | 1:100 | Mouse |
| Pan-cytokeratin | Abcam | #ab27988 | AB_777047 | AE1/AE3 | 1:500 | Mouse |
| PD-L1 | Cell Signaling | #13684 | AB_2687655 | E1L3N | 1:5,000 | Rabbit |
| Phosphor-ƴH2AX | Novus Biologicals | #NB100-2280 | AB_577933 | Polyclonal | 1:10,000 | Rabbit |
| Phospho-histone H3 | Cell Signaling | #9701 | AB_331535 | Polyclonal | 1:10,000 | Rabbit |
| Phosphor-IRF3 | Cell Signaling | #29047 | AB_2773013 | D6O1M | 1:2,500 | Rabbit |
| **Secondary antibodies** | | | | | | |
| Anti-Mouse IgG conjugated to HRP | Jackson ImmunoResearch | #115-035-003 | AB_10015289 | Polyclonal | 1:400 | Goat |
| Anti-Rabbit IgG conjugated to HRP | Jackson ImmunoResearch | #111-035-144 | AB_2307391 | Polyclonal | 1:400 | Goat |

Abbreviations: IgG, immunoglobulin; HRP, horseradish peroxidase; PD-L1, programmed death cell-ligand 1; RRID, research resource identifier.
