# Supplementary material for: In Situ Proinflammatory Effects of Dazostinag Alone or with Chemotherapy on the Tumor Microenvironment of Patients with Head and Neck Squamous Cell Carcinoma
Source: Cancer Res Commun. 2025 Jul 30;5(7):1243–55. doi: 10.1158/2767-9764.CRC-25-0314 (PMC12308172; doi:10.1158/2767-9764.CRC-25-0314)
Supplement: Supplementary Table S2 — Table S2. Probes used for in situ hybridization. [file crc-25-0314_supplementary_table_s2_suppst2.docx]

### Supplementary Table S2. Probes used for *in situ* hybridization.

| Probe | Source | Identifier |
| --- | --- | --- |
| CCL2 | Advanced Cell Diagnostics | #423811 |
| CXCL10 | Advanced Cell Diagnostics | #311851 |
| CXCL9 | Advanced Cell Diagnostics | #440161 |
| IFNB1 | Advanced Cell Diagnostics | #417071 |
| ISG15 | Advanced Cell Diagnostics | #467741 |

Abbreviations: IFNB1, interferon beta 1; ISG15, interferon-stimulated gene 15.
