# Supplementary material for: In Situ Proinflammatory Effects of Dazostinag Alone or with Chemotherapy on the Tumor Microenvironment of Patients with Head and Neck Squamous Cell Carcinoma
Source: Cancer Res Commun. 2025 Jul 30;5(7):1243–55. doi: 10.1158/2767-9764.CRC-25-0314 (PMC12308172; doi:10.1158/2767-9764.CRC-25-0314)
Supplement: Supplementary Table S3 — Table S3. Representativeness of the study population. [file crc-25-0314_supplementary_table_s3_suppst3.docx]

### Supplementary Table S3. Representativeness of the study population.

| **Cancer type** | **Head and Neck Squamous Cell Carcinoma (HNSCC): group of malignancies, involving the oral cavity, pharynx, hypopharynx, larynx, nasal cavity, and salivary glands** |
| --- | --- |
| **Considerations related to:** | **Details** |
| **Sex** | - HNSCC is approximately three times more common in males than in females globally - For malignancies in the lip and oral cavity, the male-to-female ratio is approximately 2; however, for malignancies in the larynx, the male-to-female ratio is over 7 |
| **Age** | - In the US, the median age at diagnosis is 64 years, with approximately 50% of patients diagnosed between 55 and 74 years of age |
| **Race/Ethnicity** | - In the US, among males, the incidence of HNSCC is highest in non-Hispanic White (20.1/100,000) and American Indian and Alaska Native (17.5/100,000) patients, and lowest in Hispanic (10.3/100,000) and Asian/Pacific Islander (12.0/100,000) patients - Among US females, the incidence is highest in non-Hispanic White (7.1/100,000) and Asian/Pacific Islander (5.0/100,000) patients, and lowest in Hispanic (4.3/100,000) and Black (5.0/100,000) patients |
| **Geography** | - Globally, HNSCC is more prevalent in South and Southeast Asia, followed by Central and Eastern Europe, and South America - India has the highest incidence rates due to the widespread use of tobacco, which accounts for up to 80% of all cases |
| **Other considerations** | - Tobacco use is associated with approximately 75% of HNSCC cases in Western Europe, representing the leading risk factor in this region - Human papilloma virus (HPV) infection accounts for approximately three quarters of all cases of HNSCC in developed countries versus approximately 10% of cases in developing countries - Alcohol consumption among non-smokers is associated with 4% of HNSCC cases globally |
| **Overall representativeness of this study** | - The median age of patients in the present study was 64, in line with the median age at diagnosis of HNSCC in the US - Among all 15 patients in the present study, 14 were male, consistent with the higher incidence of HNSCC in males versus females - Nine of 15 patients were smokers or previous smokers, consistent with tobacco use as a leading risk factor for HSNCC - Ten of 15 patients were HPV-positive, in line with the observed proportion of HPV-related disease in developed countries |

References:

Barsouk A, Aluru JS, Rawla P, Saginala K, Barsouk A. Epidemiology, Risk Factors, and Prevention of Head and Neck Squamous Cell Carcinoma. *Medical Sciences* 2023;**11**:42 doi:10.3390/medsci11020042

Bray F, Laversanne M, Sung H, et al. Global cancer statistics 2022: GLOBOCAN estimates of incidence and mortality worldwide for 36 cancers in 185 countries. *CA Cancer J Clin* 2024;**74**:229–63 doi:10.3322/caac.21834
