# Supplementary material for: In Situ Proinflammatory Effects of Dazostinag Alone or with Chemotherapy on the Tumor Microenvironment of Patients with Head and Neck Squamous Cell Carcinoma
Source: Cancer Res Commun. 2025 Jul 30;5(7):1243–55. doi: 10.1158/2767-9764.CRC-25-0314 (PMC12308172; doi:10.1158/2767-9764.CRC-25-0314)
Supplement: Supplementary Table S4 — Table S4. Number of regions of interest (ROIs) analyzed for each patient and drug condition in the boxplots shown in Figures 3, 4, and 5. [file crc-25-0314_supplementary_table_s4_suppst4.docx]

### Supplementary Table S4. Number of regions of interest (ROIs) analysed for each patient and drug condition in the boxplots shown in Figures 3, 4, and 5.

|  | Number of ROIs per drug condition | | | | | |
| --- | --- | --- | --- | --- | --- | --- |
| Patient | Background | Dazostinag | CPPT+PTX | CPPT+5-FU | Dazostinag+CPPT+PTX | Dazostinag+CPPT+5-FU |
| 2 | 3 | 2 | 2 |  | 3 |  |
| 3 | 3 | 3 | 3 |  | 3 |  |
| 5 | 4 | 2 | 4 | 2 | 2 | 4 |
